# ﻿Stingless bee classification and biology (Hymenoptera, Apidae): a review, with an updated key to genera and subgenera

**DOI:** 10.3897/zookeys.1172.104944

**Published:** 2023-07-27

**Authors:** Michael S. Engel, Claus Rasmussen, Ricardo Ayala, Favízia F. de Oliveira

**Affiliations:** 1 Division of Invertebrate Zoology, American Museum of Natural History, Central Park West at 79; 2 th; 3 Street, New York, NY 10024-5192, USA; 4 Department of Agroecology, Section for Entomology and Plant Pathology, Forsøgsvej 1, 4200 Slagelse, Denmark; 5 Estación de Biología Chamela, Instituto de Biología, Universidad Nacional Autónoma de México (UNAM), Apartado Postal 21, San Patricio, Jalisco 48980, Mexico; 6 Laboratório de Bionomia, Biogeografia e Sistemática de Insetos (BIOSIS), Instituto de Biologia (IBIO), Universidade Federal da Bahia (UFBA), Rua Barão de Jeremoabo, número 668, Campus Universitário de Ondina, CEP: 40170-115, Salvador, Bahia, Brazil

**Keywords:** Anthophila, Apoidea, biodiversity, biology, checklist, identification, Meliponini, phylogeny

## Abstract

Stingless bees (Meliponini) are a ubiquitous and diverse element of the pantropical melittofauna, and have significant cultural and economic importance. This review outlines their diversity, and provides identification keys based on external morphology, brief accounts for each of the recognized genera, and an updated checklist of all living and fossil species. In total there are currently 605 described extant species in 45 extant genera, and a further 18 extinct species in nine genera, seven of which are extinct. A new fossil genus, *Adactylurina* Engel, **gen. nov.**, is also described for a species in Miocene amber from Ethiopia. In addition to the systematic review, the biology of stingless bees is summarized with an emphasis on aspects related to their nesting biology and architecture.

## ﻿Dedication

*We dedicate this small contribution to the memory of three titans of the Meliponini who we had the great pleasure of knowing: Jesus S. Moure (1912–2010), Charles D. Michener (1918–2015), and João M.F. de Camargo (1941–2009). Their monumental efforts toward revealing the fundamentals of stingless bee biology, morphology, phylogeny, and evolution will never be surpassed. All of our work builds on their strong foundation.*

*In addition, we dedicate this work to our dear friend and colleague Fernando A. Silveira (1960–2022), whose untimely passing in August 2022 deprived melittology of one of its kindest and most generous scholars. He is greatly missed.*

## ﻿Introduction

**Figure 1.**
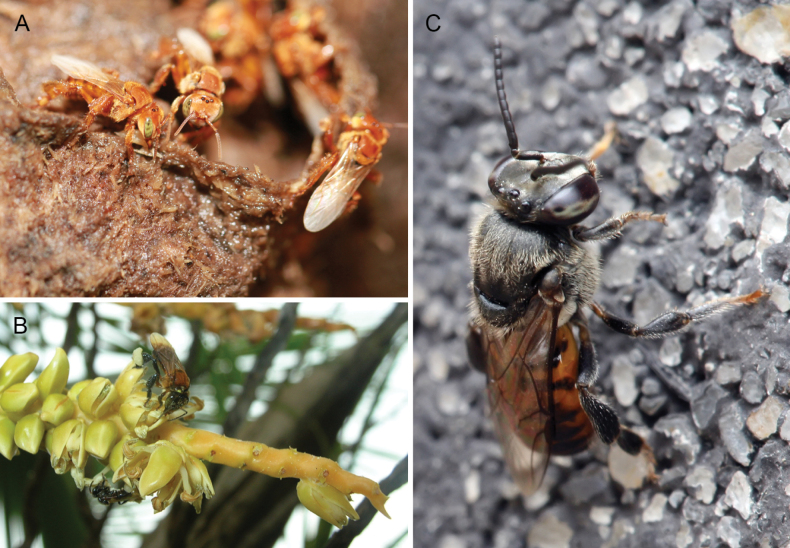
Representative stingless bees from three biogeographic realms **A** workers of Trigona (Trigona) dallatorreana Friese from Peru (photograph C. Rasmussen) **B** workers of *Geniotrigonalacteifasciata* (Cameron) from Malaysia (photograph C. Rasmussen) **C** male of Axestotrigona (Axestotrigona) ferruginea (Lepeletier) from Tanzania (photograph Muhammad Mahdi Karim, Wikimedia Commons, GNU Free Documentation License, Version 1.2: https://commons.wikimedia.org/wiki/Commons:GNU_Free_Documentation_License,_version_1.2).

In the tropical and subtropical environs of the world, one of the predominant lineages of social bees is the tribe Meliponini (Fig. 1). They are popularly known as indigenous bees or stingless bees due to the atrophy of the sting, which is no longer functional as a defensive weapon. At around 605 species the stingless bees are the most diverse lineage of the corbiculate bees, a clade that includes the most iconic groups of bees throughout the world: honey bees (Apini), bumble bees (Bombini), orchid bees (Euglossini), and, of course, the stingless bees (Meliponini). They are managed for their honey, second only to the honey bees, and are growingly used for agricultural purposes (e.g., Heard 1999; Slaa et al. 2006; Jha and Dick 2010). Meliponiculture, just like apiculture and the burgeoning area of bombiculture, is a growing industry in tropical countries and aside from pollination services, bee products such as honey, propolis, resin, and collected pollen are all key to human food, health, and food security. Stingless bees, like honey bees, are also key to the cultural and religious practices of many ancient and current indigenous peoples, further emphasizing how the bees are key to the everyday lives of those living in the tropics.

The purpose of this chapter is to briefly summarize the phylogeny and evolution, current classification, and general biology of stingless bees. Naturally, these subjects could occupy entire books in their own right and it is therefore impossible for any of these topics to be afforded sufficient justice or depth as to satisfy most readers. Therefore, the present effort merely attempts to whet the appetite of the mind and direct the reader to where more thorough information may be sought. In this regard, we would be remiss if we did not mention the recent and excellent tome by Christoph Grüter that covers the biology, evolution, and ecology of Meliponini in greater depth than we could ever hope (Grüter 2020). Beyond this book, excellent reviews are by Michener (2007a, 2013), Melo (2021), and Quezada-Euán (2018), and although now somewhat dated the review of meliponine nest architecture by Wille and Michener (1973), the review of stingless bee sociobiology by Michener (1974), and those on their biology and evolution by Schwarz (1948), Wille (1979, 1983), and Roubik (2023) all remain indispensable resources.

## ﻿Phylogeny and evolution

**Figure 2.**
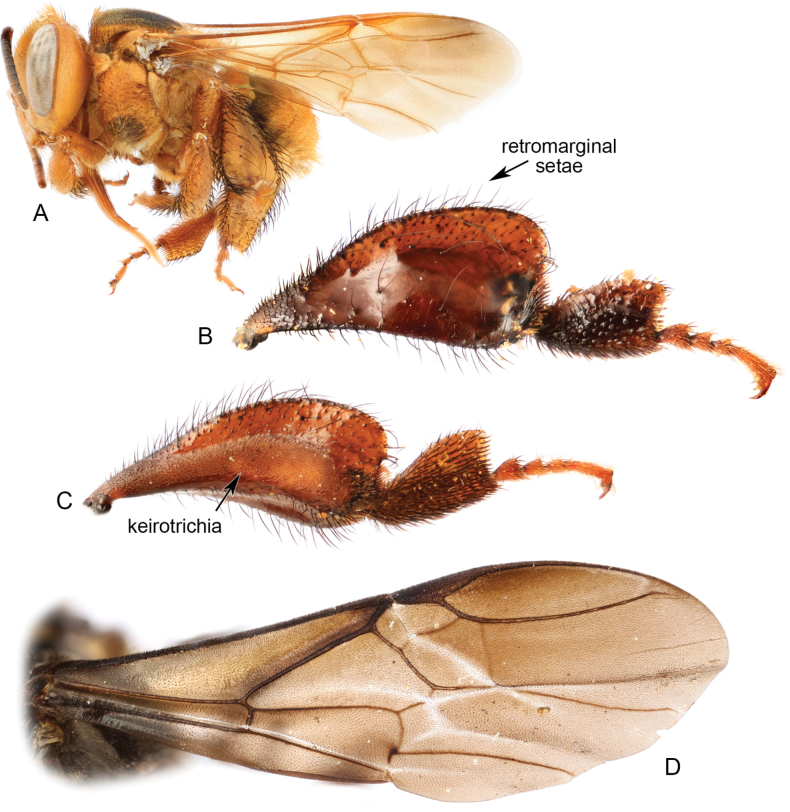
Details of meliponine morphology **A** lateral habitus of worker of Scaptotrigona (Scaptotrigona) magdalenae Engel from Colombia **B** prolateral surface of metatibia and metarsus of *Cephalotrigonazexmeniae* (Cockerell) from Guatemala **C** retrolateral surface of metatibia and metarsus of *C.zexmeniae* from Guatemala **D** forewing of *Wallacetrigonaincisa* (Sakagami & Inoue) from Sulawesi. Individual images from Engel (2022c), Engel et al. (2021a), and Rasmussen et al. (2017). All images M.S. Engel (used with permission).

Stingless bees are a long-recognized lineage of apine in the superfamily Apoidea, and belong to the clade of corbiculate tribes. The corbiculate bees are so named for the possession of a metatibial corbicula in females of non-parasitic forms (Fig. 2). In common parlance the corbicula is the proverbial “pollen basket”, used for the transport of pollen wetted by nectar and saliva. The structure consists of a broadened, depressed, smooth, largely glabrous area on the apical prolateral surface of the metatibia, typically fringed by long setae that help to create and define a negative space in which the resources are held. Aside from pollen, the corbicula can be used to transport other materials, such as resins or mud.

The Meliponini are universally recognized as a monophyletic group, supported by a large number of specializations relative to other corbiculate bees. Some of the key traits distinguishing Meliponini include: the presence of a penicillum, lack an auricle (“pollen press”) proximally on the metabasitarsus, the simple pretarsal claws, alar venation reduction, and, of course, the general reduction of sclerites associated with the sting complex. Other features that in combination help to define Meliponini but are found in other combinations among the remaining corbiculate tribes are the general reduction of outer grooves on the mandibles, the loss of the metatibial spurs, the presence of arolia, the absence of a supra>-alar carina, and the presence of a jugal lobe on the hind wing (Michener 1990, 2007a; Engel et al. 2021a, 2021b; Engel and Rasmussen 2021). Systematic coverages of meliponine immature stages have been provided by Rozen (2021), Rozen et al. (2019a, 2019b, 2021), and Rozen and Smith (2019).

Naturally, one of the hallmark traits of stingless bees is the largely vestigial sting apparatus. The various structures associated with the sting complex are present in Meliponini, but they are reduced and generally nonfunctional, although those of the African *Meliponula* Cockerell are comparatively well developed along with an enlarged poison gland (Kerr and Lello 1962), suggesting the potential for some minor functionality as a defensive apparatus. The reduction of the sting perhaps reflects a history in which the lineage underwent significant body size diminution, a “miniaturization bottleneck”, with a sting no longer serving as an effective deterrent for vertebrate predators (Wille 1979). This has been lent some added credence by the early diverging position of *Trigonisca* Moure, a group of entirely minute stingless bees (Rasmussen and Cameron 2010), as well as the general loss of wing venation concomitant with a more enlarged pterostigma, which is another suite of modifications found among minute insects. While the sting was rendered inert, the nests of stingless bees gained considerably in their defensive qualities, picking up where the potency of a sting was removed. Nonetheless, while vestigial sting sclerites are distinctive for meliponines and inaccessible and tough nest architectures prevent many predators from interfering, stingless bees are not defenseless (Roubik 2023).

The closest relatives of Meliponini are the species of the extinct tribe Melikertini (Engel 1998, 2001a, 2001b; Schultz et al. 2001), a group of corbiculate bees that disappeared around the time of the Eocene-Oligocene mass extinction event, approximately 35 million years ago (Engel 2001a, 2001b; Engel and Davis 2021). Melikertines were globally distributed and seemingly tropical, subtropical, and paratropical eusocial bees, but unlike Meliponini possessed the complete complement of wing venation, had a well-developed and insertable sting, possessed a functional pollen press, and had metatibial spurs, albeit quite reduced relative to those of other bees (Engel and Davis 2021). Like Meliponini, melikertines seem to have commonly collected resin, presumably to be used similarly in nest construction (Engel and Davis 2021). Melikertines and meliponines coexisted for a considerable period of history before the disappearance of the former at the end of the Eocene.

**Figure 3.**
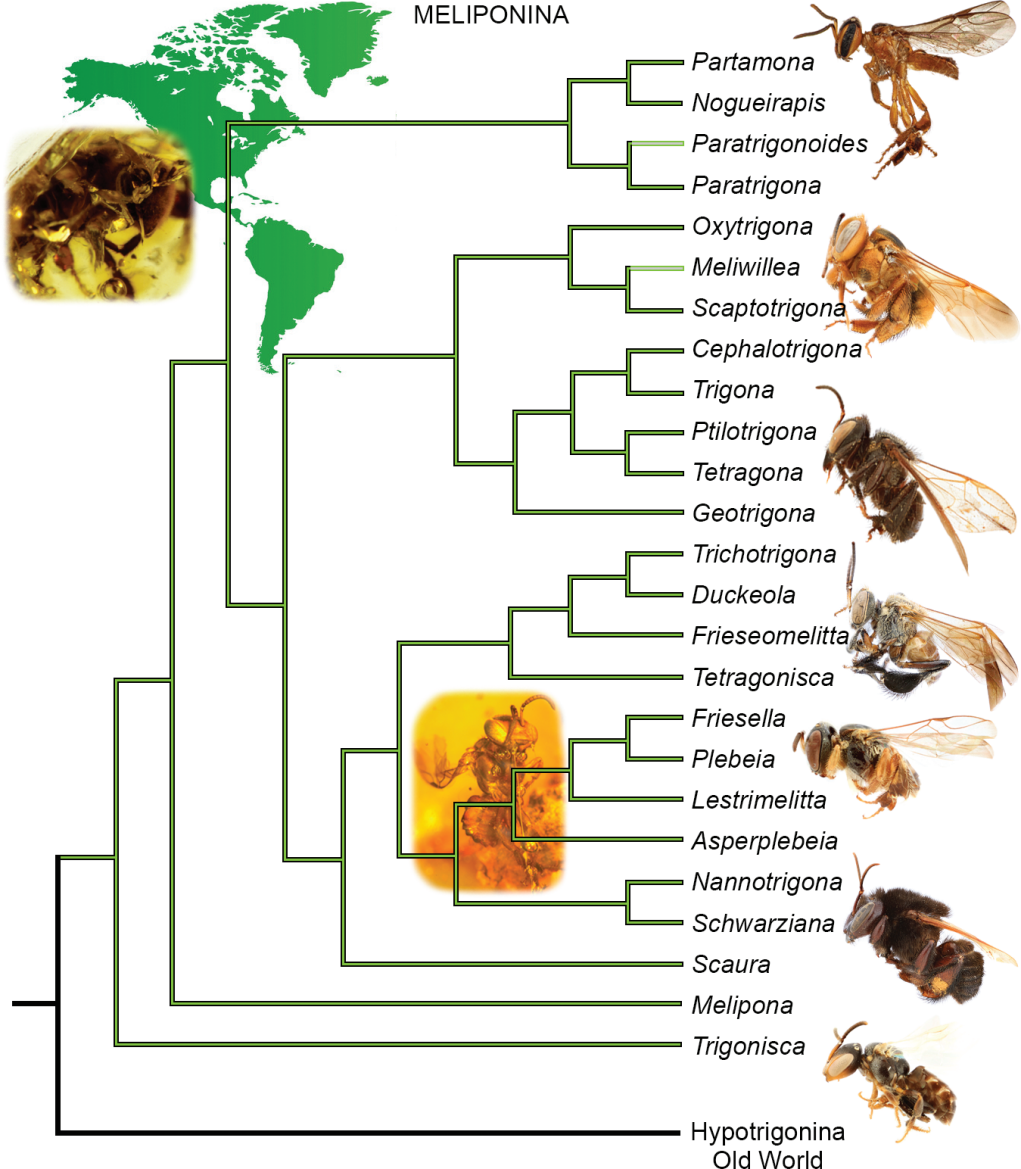
Phylogeny of subtribe Meliponina (New World Meliponini), summarized from Rasmussen and Cameron (2010), with hypothesized placements of *Paratrigonoides* Camargo & Roubik and *Meliwillea* Roubik, Lobo Segura, & Camargo. Representative bees at right (from top to bottom, not to same scale): *Nogueirapismirandula* (Cockerell), Scaptotrigona (Scaptotrigona) magdalenae Engel, Trigona (Necrotrigona) crassipes (Fabricius), *Frieseomelittatrichocerata* Moure, Plebeia (Nanoplebeia) pleres Engel, Melipona (Mouremelia) fuliginosa Lepeletier, Trigonisca (Trigonisca) mepecheu Engel & Gonzalez. Representative fossil bees (from top to bottom, not to same scale): *Cretotrigonaprisca* (Michener & Grimaldi) in Maastrichtian New Jersey amber, *Proplebeiasilacea* (Wille) in Miocene Chiapas amber. Images of fossil bees from Engel (2000a) and Engel et al. (2021a). All images M.S. Engel (used with permission).

**Figure 4.**
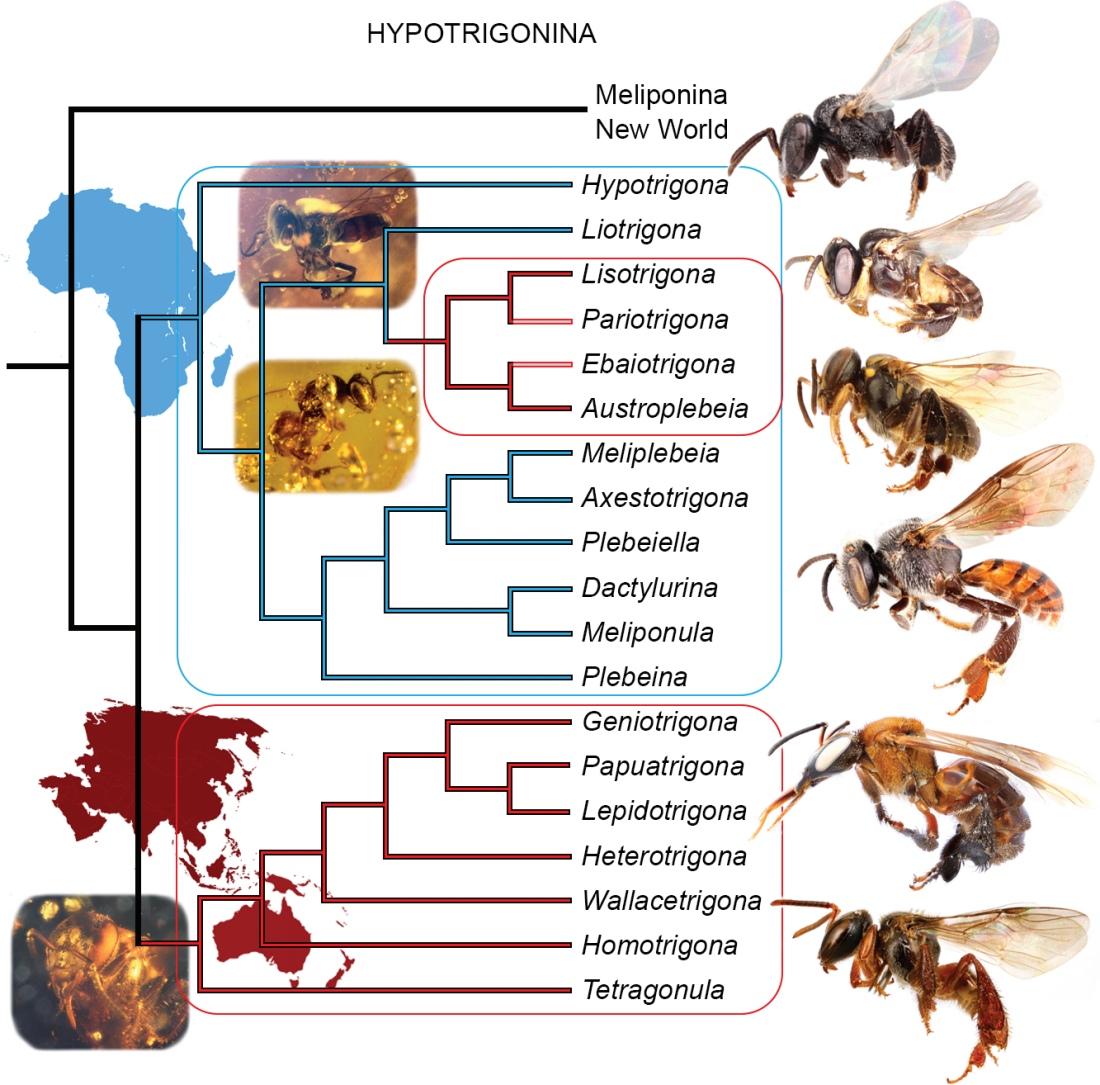
Phylogeny of subtribe Hypotrigonina (Old World Meliponini), summarized from Rasmussen and Cameron (2010), with hypothesized placements of *Pariotrigona* Moure and *Ebaiotrigona* Engel & Nguyen. Blue branches indicate African lineages, while red branches indicate Southeast Asian-Malesian-Papuasian-Australian lineages. Representative bees at right (from top to bottom, not to same scale): *Hypotrigonagribodoi* (Magretti), *Ebaiotrigonacarpenteri* (Engel), Austroplebeia (Austroplebeia) cincta (Mocsáry), *Plebeinaarmata* (Magretti), *Geniotrigonalacteifasciata* (Cameron), Tetragonula (Tetragonula) malaipanae Engel, Michener, & Boontop. Representative fossil bees (from top to bottom, not to same scale): Liotrigona (Tapheiotrigona) aethiopica Engel in Miocene Ethiopian amber, *Liotrigonopsisrozeni* Engel in Eocene Baltic amber, Tetragonula (Tetragonula) florilega Engel in Miocene Zhangpu amber. Images of bees from Engel et al. (2021a), Engel and Aber (2022), Engel (2001a), and Rasmussen et al. (2017). All images M.S. Engel (used with permission).

There has been considerable interest in the phylogeny of stingless bees, not only in terms of their classification but also for understanding their biogeography, behavior, physiology, and nest architecture, among other phenomena. An understanding of relationships among the lineages of Meliponini has shifted considerably over the years. Earlier authors presented a wide number of hypotheses, typically with small differences but sometimes wholly incompatible, all based on different interpretations or analyses of morphological and/or biological data (e.g., Wille 1983), although some also incorporated or were based upon small swaths of DNA sequences (e.g., Costa et al. 2003). More recently, however, a detailed and comprehensive exploration of molecular data has helped to provide some greater clarity, and a pattern of overall relationships that is robust and simultaneously consistent with several morphological, behavioral, and biogeographic patterns (Rasmussen and Cameron 2010). These relationships, in turn, have helped to refine elements of the classification as well as formulate new hypotheses regarding meliponine evolution and are summarized in Figs 3, 4.

### ﻿Classification

For more than half of the time since the start of formal binomial nomenclature in 1758, the stingless bees were largely classified, with some minor exceptions (e.g., the earliest species were placed in *Apis* Linnaeus or *Centris* Fabricius), in either a single genus, *Melipona*, or into two genera, *Melipona* Illiger and *Trigona* Jurine. It was not until the works of Heinrich Friese (1860–1948) and Theodore D.A. Cockerell (1866–1948) that serious alterations to the classification were initiated, although most species were still placed in a massive, ill-circumscribed *Trigona*. These two authors were then followed by the extensive and detailed studies of Herbert F. Schwarz (1883–1960) and Jesus S. Moure (1912–2010), who effectively provided new interpretations for natural groupings of stingless bees, with the latter establishing the foundations for our current system of Meliponini. The growing biological data available for many species also served as a new source of character information for establishing groupings of these bees (e.g., Schwarz 1948). Subsequent to Moure, most changes to the supraspecific classification consisted of the addition of new taxa, largely through the efforts of João M.F. Camargo (1941–2009) and his collaborators in the New World and Shôichi F. Sakagami (1927–1996) in the Old World (e.g., Sakagami 1975, 1978, 1982). Contra these systems, Charles D. Michener (1918–2015) (Michener 1990, 2007a) and Alvaro Wille (1928–2006) (Wille 1963, 1979) tended to reduce the number of recognized genera, but the phylogenies of Rasmussen and Cameron (2007, 2010) corroborated many of the conclusions originally raised by Moure (1946, 1951, 1961, 1971), thereby necessitating a considerable reconsideration of their organization.

Here we present a brief overview of the revised supraspecific classification of Meliponini (M.S. Engel in Engel et al. 2021a), including keys to the genera and subgenera by region and summaries of distribution and identification tools for each genus, along with a checklist of currently recognized species. The three keys presented here allow for the identification of all currently recognized genera and subgenera of Meliponini. The keys are adapted from recent works that relate to the new organization of the tribe (Rasmussen et al. 2017; Engel 2019; Engel et al. 2021a) and are organized by geographic region, in the same manner as was done by Michener (2007a), although with a considerably different arrangement of genera. Naturally, there will always be disagreement over the recognition of particular groups and at what rank they should be classified, and different systematists will advocate for slightly different systems depending on their concepts of the groups. Even the authors of this chapter do not agree on all points of the current classification (e.g., whether *Leurotrigona* Moure should be removed from *Trigonisca* as its own genus, whether Dolichotrigona Moure should be recognized as a valid subgenus independent of Trigonisca s.str., whether *Mourella* Schwarz should be accorded generic rank, etc.). These finer points are all worthy of merit and undoubtedly will be revised in time. For now, we have followed the system as recently outlined in Engel et al. (2021a), with the exception of elevating *Mourella* as a genus and some additions from the last year (e.g., subgenera of *Geotrigona* Moure, *Scaptotrigona* Moure, and *Scaura* Schwarz) and herein (one new genus). Although we allude to some disparities and future subjects to be resolved, we do not intend here to emphasize justification or alternative interpretations, which are more fully explained in some of the recent publications cited herein. Regardless, the revised classification is based on comprehensive phylogenetic hypotheses for the tribe (Rasmussen and Cameron 2010).

Morphological terminology for the keys generally follows that used in major works on bees (e.g., Engel 2001a; Michener 2007a), although the terms of orientation for the legs follow that outlined by Engel et al. (2021a) and wing venation terms are from Rasmussen et al. (2017). Following the keys are brief summaries for each genus, along with a list of those currently recognized species in each taxon (subspecies and synonyms are not listed; species lists are current as of 29 June 2023).

### ﻿Neotropical Meliponini

In the New World, stingless bees are found from 34.89°S in Uruguay (Montevideo) and Argentina (Buenos Aires) up to 27.03°N in Mexico (Álamos, Sonora), at exceptional elevations up to 4000 m.a.s.l. in Peru and Bolivia (Camargo and Pedro 2007; Roig-Alsina and Alvarez 2017; Roubik and Vergara 2021). Meliponini are not native elsewhere in the Greater or Lesser Antilles, although extinct species of the genus *Proplebeia* Michener are known from the Miocene of the Dominican Republic and Chiapas, México. Camargo and Pedro (2007) and Camargo et al. (2013) provided a catalog of the Neotropical Meliponini. All Neotropical stingless bees belong to the subtribe Meliponina (Rasmussen and Cameron 2010; Engel et al. 2021a), a group not found in other regions of the world (Table 1). As of this writing we recognize 474 extant species in the New World and 26 extant genera.

**Table 1.** Hierarchical classification of Western Hemisphere stingless bees (Meliponini: Meliponina).

Subtribe **Meliponina** Lepeletier
[New World Meliponini]
Infratribe Meliponitae Lepeletier
*Paratrigona* Genus Group
Genus *Paratrigona* Schwarz, s.l.
Subgenus Aparatrigona Moure
Subgenus Paratrigona Schwarz, s.str.
Genus *Paratrigonoides* Camargo & Roubik
Genus *Nogueirapis* Moure
Genus *Partamona* Schwarz, s.l.
Subgenus Partamona Schwarz, s.str.
Subgenus Parapartamona Schwarz
*Trigona* Genus Group
Genus *Oxytrigona* Cockerell
Genus *Scaptotrigona* Moure
Subgenus Eoscaptotrigona Engel
Subgenus Sakagamilla Moure
Subgenus Gymnotrigona Engel
Subgenus Astegotrigona Engel
Subgenus Baryorygma Engel
Subgenus Dasytrigona Engel
Subgenus Scaptotrigona Moure, s.str.
Genus *Meliwillea* Roubik, Lobo Segura, & Camargo
Genus *Geotrigona* Moure, s.l.
Subgenus Chthonotrigona Engel
Subgenus Geotrigona Moure, s.str.
Genus *Ptilotrigona* Moure, s.l.
Subgenus Camargoia Moure
Subgenus Ptilotrigona Moure, s.str.
Genus *Tetragona* Lepeletier & Audinet-Serville
Genus *Trigona* Jurine, s.l.
Subgenus Aphaneuropsis Engel
Subgenus Koilotrigona Engel
Subgenus Necrotrigona Engel
Subgenus Nostotrigona Engel
Subgenus Ktinotrofia Engel
Subgenus Aphaneura Gray
Subgenus Trigona Jurine, s.str.
Subgenus Dichrotrigona Engel
Genus *Cephalotrigona* Schwarz
*Plebeia* Genus Group
Genus *Tetragonisca* Moure
Genus *Frieseomelitta* Ihering
Genus *Trichotrigona* Camargo & Moure
Genus *Duckeola* Moure
Genus *Plebeia* Schwarz, s.l.
Subgenus Nanoplebeia Engel
Subgenus Plebeia Schwarz, s.str.
Genus *Lestrimelitta* Friese
Subgenus Apiraptor Engel
Subgenus Hyrolestris Engel
Subgenus Lestrimelitta Friese, s.str.
Genus *Friesella* Moure
Genus *Asperplebeia* Engel
Genus *Nannotrigona* Cockerell, s.l.
Subgenus Lispotrigona Gonzalez & Engel
Subgenus Nannotrigona Cockerell, s.str.
Genus *Mourella* Schwarz
Genus *Schwarziana* Moure, s.l.
Subgenus Chapadapis Engel
Subgenus Schwarziana Moure, s.str.
Genus *Scaura* Schwarz, s.l.
Subgenus Scaura Schwarz, s.str.
Subgenus Scauracea Engel
Subgenus Schwarzula Moure
Genus †*Proplebeia* Michener
*Melipona* Genus Group
Genus *Melipona* Illiger, s.l.
Subgenus Melipona Illiger, s.str.
Subgenus Meliponiella Melo
Subgenus Melikerria Moure
Subgenus Eomelipona Moure
Subgenus Mouremelia Engel
Subgenus Michmelia Moure
Infratribe Trigoniscitae Engel
*Trigonisca* Genus Group
Genus *Trigonisca* Moure, s.l.
Subgenus Leurotrigona Moure
Subgenus Exochotrigona Engel
Subgenus Celetrigona Moure
Subgenus Trigonisca Moure, s.str.
Genus †*Exebotrigona* Engel & Michener
Subtribe *Incertae sedis*
Genus †*Cretotrigona* Engel [Meliponina?]

## ﻿Results

### ﻿Key to genera and subgenera of Neotropical Meliponini (expanded and modified from Michener 2007a; Engel et al. 2021a; Engel 2021d, 2022d)

**Table:** 

1	Base of marginal cell broad, basal angle (within marginal cell between pterostigmal margin and r-rs) slightly acute (not under 68°) to orthogonal; marginal cell, at apex of pterostigma, broader than submarginal cell area; forewing less, usually much less, than 4 mm long [genus *Trigonisca* Moure, s.l.]	**2**
–	Base of marginal cell comparatively narrow, basal angle strongly acute (≤ 50°) (except ~ 80° in *Nogueirapis* Moure); marginal cell, at apex of pterostigma, little if any broader than submarginal cell area; forewing usually > 4 mm long	**5**
2(1)	Integument of mesoscutum and mesoscutellum smooth and shiny; preoccipital carina absent; transscutal sulcus between axillae shallowly impressed; mesoscutellum comparatively flat and low, acutely rounded apically in profile and slightly raised above level of metanotum	**3**
–	Integument of mesoscutum and mesoscutellum matte, microalveolate to tessellate; preoccipital carina present at least laterally, sometimes weakly so; transscutal sulcus between axillae deeply and broadly impressed; mesoscutellum gently convex, broadly rounded apically in profile and distinctly raised above level of metanotum	**4**
3(2)	Malar space as long as 2× flagellar diameter; retrodorsal margin of metatibia gently arched, without projection at superior distal angle; superior parapenicillum curved but not greatly sinuate; head width ≥ 1.0 mm	**Trigonisca (Leurotrigona) Moure**
–	Malar space as long as flagellar diameter; retrodorsal margin of metatibia somewhat sinuous, with superior distal angle projected; superior parapenicillum markedly sinuate; head width < 1.0 mm	**Trigonisca (Exochotrigona) Engel**
4(2)	Labrum simple; setae along retrodorsal margin of metatibia as long as or shorter than maximum metatibial width	**Trigonisca (Trigonisca) Moure**
–	Labrum bituberculate; setae along retrodorsal margin of metatibia distinctly longer than maximum metatibial width	**Trigonisca (Celetrigona) Moure**
5(1)	Retrolateral surface of metatibia with strongly depressed, shiny, superior marginal subglabrate zone, which at least apically is usually approximately as broad as longitudinal median keirotrichiate ridge, and midway of metatibial length is at least half as wide as keirotrichiate ridge	**6**
–	Retrolateral surface of metatibia with depressed superior marginal subglabrate zone narrower (much less than half as wide as area with keirotrichia) or absent, keirotrichia extending to or close to margin	**24**
6(5)	Compound eyes with inconspicuous setae; rastellum strongly developed	**7**
–	Compound eyes setose; rastellum reduced to tapering setae	***Trichotrigona* Camargo & Moure**
7(6)	Face of ordinary shape, minimum distance between compound eyes little more than to less than length of compound eye; clypeus usually > 2× as broad as long; malar space slightly > 1.5× as long as flagellar diameter or usually much less; keirotrichiate zone on retrolateral surface of worker metatibia usually narrow, rarely > 1.5× as wide as depressed superior marginal subglabrate zone at midlength of metatibia	**8**
–	Face short and broad, minimum distance between compound eyes much greater than length of compound eye; clypeus < 2× as broad as long; malar space almost 2× as long as flagellar diameter; keirotrichiate zone on retrolateral surface of worker metatibia nearly twice as wide as depressed superior marginal subglabrate zone at midlength of metatibia	***Oxytrigona* Cockerell**
8(7)	Preoccipital carina absent; lower face and genal area finely sculptured like upper part of head and mesoscutum	**9**
–	Preoccipital carina strong and shiny across full width behind vertex; lower face and genal area shiny and coarsely punctate in contrast to dull, densely, minutely punctate upper face, genal area, and mesoscutum	***Cephalotrigona* Schwarz**
9(8)	Mandible of worker with 4 or 5 teeth along distal margin; retrolateral surface of metabasitarsus of males and workers with basal sericeous area [genus *Trigona* Jurine, s.l.]	**10**
–	Mandible of worker with lower half or 2/3 of distal margin edentate, upper part of margin with 1 or usually 2 teeth; retrolateral surface of metabasitarsus of males without basal sericeous area, that of workers, variable	**17**
10(9)	Mandible with 4 teeth	**11**
–	Mandible with 5 teeth	**12**
11(10)	Labrum simple, surface gently and evenly convex; wings dichroic, proximally infuscate, apically whitish; pterostigma yellowish brown; scape black	**Trigona (Aphaneuropsis) Engel**
–	Labrum bigibbous, with pronounced mediolongitudinal furrow; wings uniformly fuscous; pterostigma brown to dark brown; scape paler ventrally, pale brown to yellowish brown	**Trigona (Koilotrigona) Engel**
12(10)	Metatibia with distinct corbicula on apical prolateral surface (corbicular surface concave), superior distal angle present, retromarginal fringe setae abundant	**13**
–	Metatibia without defined corbicula on apical prolateral surface (corbicular surface not concave), superior distal margin rounded, retromarginal fringe setae less numerous	**Trigona (Necrotrigona) Engel**
13(12)	Labrum simple, surface gently and evenly convex; vertex with distinct postocellar ridge; integument entirely dark brown to black (except entirely orange in *Trigonadallatorreana* Friese)	**14**
–	Labrum bigibbous, with mediolongitudinal furrow (furrow somewhat weak in *T.williana* Friese); vertex without postocellar ridge, or ridge quite weak; integument largely yellowish orange (head, mesoscutum, and parts of pleura sometimes largely black but clypeus and antenna always yellowish to yellowish orange)	**Trigona (Aphaneura) Gray**
14(13)	Wing membrane not as below, if slightly paler apically, then transition gradual across wing length; metatibial width variable, sometimes comparatively narrow	**15**
–	Wing membrane strikingly dichroic, proximally darkly infuscate, apically whitish; metatibia broad apically, with broadly rounded retromarginal contour	**Trigona (Dichrotrigona) Engel**
15(14)	Small bees, head width ≤ 2.5 mm; forewing (including tegula) length < 6.5 mm; metatibia narrow, retromarginal contour comparatively straight until apical fifth	**16**
–	Larger bees, head width typically ≥ 2.5 mm or greater, rarely as small as 2.45 mm (in some *T.corvina* Cockerell), forewing (including tegula) length ≥ 6.9 mm; metatibia broader, typically with broadly rounded retromarginal contour	**Trigona (Trigona) Jurine**
16(15)	Apical fundal surface of metatibia near corbicula with abundant minute, fine, appressed setae; scape with prominent, thick, black, bristle-like setae along length, such setae often as long as scape diameter; clypeus in profile with numerous, erect, black, bristle-like setae; distance from median ocellus to postocellar ridge about ocellar diameter; smaller bees, intertegular distance ≤ 1.35 mm	**Trigona (Nostotrigona) Engel**
–	Apical fundal surface of metatibia near corbicula with minute, fine, appressed setae either lacking or exceedingly sparse; scape without thick bristle-like setae, with fine, paler setae, such setae shorter than scape diameter; clypeus in profile with a few short, fine setae, without black bristle-like setae; distance from median ocellus to postocellar ridge less than ocellar diameter; larger bees, interegular distance ≥ 1.4 mm	**Trigona (Ktinotrofia) Engel**
17(9)	Retrolateral surface of metabasitarsus of worker without basal sericeous area, rather uniformly setose	**18**
–	Retrolateral surface of metabasitarsus of worker with basal sericeous area covered with minute setae or sometimes lacking setae	***Tetragonisca* Moure**
18(17)	Mesotibial spur absent; gena with sparse setation, not obscuring integument; mandibular teeth small denticles; profundal surface with setation variable, typically with numerous plumose setae amid simple, erect, setae; M+Cu in line with 1Cuα and 1Cuβ, or, if 1Cuα more transverse, then 1Cuβ offset from M+Cu by vein width	**19**
–	Mesotibial spur present; gena with dense setation, with overall velvety appearance; mandibular teeth typically strong and prominent; profundal surface with sparse plumose setae amid simple, erect, setae; M+Cu distinctly offset from 1Cuβ, with 1Cuα nearly transverse and 1Cuβ offset by more than vein width and superficially appearing as if arising from 1cu-a	**22**
19(18)	Metasoma short, about as wide as mesosoma, dorsoventrally flattened; retrodorsal margin of metatibia of worker usually with few plumose setae, of those, most with only 2–6 scattered branches not concentrated toward apices; yellow markings absent, integument brown to black; vein M of forewing dark almost to wing margin; discs of metasomal sterna with abundant, erect setae, some with curved apices [genus *Geotrigona* Moure, s.l.]	**20**
–	Metasoma usually narrower than mesosoma, often noticeably elongate; retrodorsal margin of metatibia of worker with numerous plumose setae, typically with abundant branches toward apices; yellow markings always present, albeit sometimes reduced and restricted to clypeus and paraocular areas; vein M of forewing usually fading away near widest part of wing; discs of metasomal sterna with diffusely scattered, long, erect, simple setae	**21**
20(19)	Metatibia with apical margin rounding continuously to broadly rounded superior distal curve, not projected into a distinct angle or tooth before superior angle; apical margin straight or weakly concave	**Geotrigona (Geotrigona) Moure**
–	Metatibia with apical margin distinctly projecting into an angle or tooth before superior angle; apical margin, between apex of tooth and penicillum, deeply concave	**Geotrigona (Chthonotrigona) Engel**
21(19)	Posterior margin of vertex not elevated; superior distal angle of metatibia of worker broadly rounded; labial palpus with numerous elongate, sinuous setae (setae of first two palpomeres as long as palpomere I and longer than palpomere II, typically > 2× palpal width); smaller bees, typically ≤ 7 mm in length	***Frieseomelitta* Ihering**
–	Posterior margin of vertex elevated as strong, setose ridge between summits of compound eyes; superior distal angle of metatibia of worker acute; labial palpus with some long setae (setae ≤ 1.5× palpal width) on first two palpomeres, such setae apically curved or rarely sinuous; larger bees, ~ 8–9 mm in length	***Duckeola* Moure**
22(18)	Basal area of propodeum setose, sometimes with mediolongitudinal glabrous line between lateral areas of wispy setae [genus *Ptilotrigona* Moure, s.l.]	**23**
–	Basal area of propodeum wholly glabrous	***Tetragona* Lepeletier & Audinet-Serville**
23(22)	Basal area of propodeum pubescent; labial palpus with setae no longer than palpal width and straight or nearly so	**Ptilotrigona (Ptilotrigona) Moure**
–	Basal area of propodeum with mediolongitudinal glabrous line between lateral areas of wispy setae; labial palpus with long, sinuous setae	**Ptilotrigona (Camargoia) Moure**
24(5)	Prolateral surface of metatibia convex, without corbicula, proventral margin convex like retromarginal contour; penicillum absent; rastellum consisting of tapering setae; first flagellomere of worker nearly as long as combined lengths of second and third flagellomeres, that of male nearly as long as second flagellomere [genus *Lestrimelitta* Friese, s.l.]	**25**
–	Prolateral surface of metatibia flat or concave at least apically, forming corbicula, proventral margin gently convex to concave, differing from largely or wholly convex retromarginal contour; penicillum present; rastellum variable; first flagellomere of worker shorter than combined lengths of second and third flagellomeres, that of male much shorter than second flagellomere	**27**
25(24)	Propodeal spiracle ovoid	**26**
–	Propodeal spiracle elongate linear, slit-like	**Lestrimelitta (Hyrolestris) Engel**
26(25)	Propodeal spiracle with upper margin pronounced relative to lower margin; inner orbits of compound eyes parallel to faintly diverging; larger bees, total length > 5 mm	**Lestrimelitta (Lestrimelitta) Friese**
–	Propodeal spiracle with upper margin similar to lower margin; inner orbits of compound eyes weakly converging below; small bees, total length < 5 mm	**Lestrimelitta (Apiraptor) Engel**
27(24)	Hind wing with 9–14 (rarely 8) hamuli; wings extending little if any beyond apex of metasoma; pterostigma with margin within marginal cell straight or weakly concave (body apiform; basal area of propodeum dull, setose) [genus *Melipona* Illiger, s.l.]	**28**
–	Hind wing with 5–7 hamuli, rarely up to 9 or even 10; wings long, extending well beyond apex of metasoma; pterostigma with margin within marginal cell slightly convex	**33**
28(27)	Vertex posterior to ocelli at most only slightly elevated; ocellocular area comparatively flat, not depressed; pronotal posterior dorsal ridge typically present and forming a surface of smooth and rounded contour bordering mesoscutum, rarely forming a crest; lower surface of mesepisternum variable, often microreticulate and matte; mesoscutum, axilla, and mesoscutellum often with yellow maculation	**29**
–	Vertex posterior to ocelli distinctly elevated; ocellocular area depressed, forming a distinct concave surface; pronotal posterior dorsal ridge virtually absent, pronotal posterior border tightly approximating anterior border of mesoscutum and forming a sharp crest; ventral surface of mesepisternum shiny; mesoscutum, axilla, and mesoscutellum without yellow maculation	**Melipona (Melipona) Illiger**
29(28)	Mandible with comparatively small preapical teeth, separation between first (P_1_) and second (P_2_) preapical teeth a smooth arc; anterolateral areas of mesoscutum with setae similar in color to those of remainder of mesoscutum; superior apical angle of metatibial apical margin forming a short projection	**30**
–	Mandible with comparatively more prominent preapical teeth, separation between first (P_1_) and second (P_2_) preapical teeth a deep V-shaped incision; anterolateral areas of mesoscutum with dense tufts of testaceous or tawny setae contrasting with setae of remainder of mesoscutum; superior apical angle of metatibial apical margin forming a prominent projection	**Melipona (Melikerria) Moure**
30(29)	Malar space short, distinctly shorter than flagellar diameter; upper interorbital distance distinctly less than length of compound eye; interocellar distance greater than ocellocular distance	**31**
–	Malar space long, as long as or longer than flagellar diameter; upper interorbital distance equal to or slightly less than length of compound eye; interocellar distance typically shorter than ocellocular distance	**32**
31(30)	Mesoscutellum with prominent, broad yellow maculation along lateral margins, yellow maculation extending from axilla along nearly entire margin of equal width, extending anterior to tegula and nearly to pronotal lobe; clypeus slightly arched	**Melipona (Meliponiella) Melo**
–	Mesoscutellum without yellow lateral borders or, if some yellow maculation present, then broader near axilla and tapering anteriorly before disappearing entirely by middle of tegula; clypeus flat	**Melipona (Eomelipona) Moure**
32(30)	Lower half of face polished and shiny, devoid, or nearly so, of setae, contrasting with upper half of face; body length typically ≥ 12 mm	**Melipona (Mouremelia) Engel**
–	Lower half of face dull, matte, with numerous setae, and without contrasting setation between lower and upper halves of face; body length typically ≤ 11 mm	**Melipona (Michmelia) Moure**
33(27)	Anterior margin of mesoscutellum with shiny, longitudinal, V- or U-shaped median depression opening anteriorly into mesoscutal-mesoscutellar fossa; preoccipital carina present, extending far down each side of head	**34**
–	Anterior margin of mesoscutellum without shiny, longitudinal, median depression; preoccipital carina absent or weakly indicated only by transverse dorsal section posterior to vertex (except in *Paratrigonoides* Camargo & Roubik)	**42**
34(33)	Integument of head and mesosoma, or at least mesoscutellum, with coarse, cribriform punctation; posterior margin of mesoscutellum notched or emarginate medially; anterior margin of pronotal lobe with strong, transverse carina [genus *Nannotrigona* Cockerell, s.l.]	**35**
–	Integument of head and mesosoma with fine punctation; posterior margin of mesoscutellum entire; anterior margin of pronotal lobe rounded [genus *Scaptotrigona* Moure, s.l.]	**36**
35(34)	Mesoscutum and mesoscutellum with dense, coarse, cribriform punctures; larger bees, head width > 1.6 mm	**Nannotrigona (Nannotrigona) Cockerell**
–	Mesoscutum sparsely punctate, integument between punctures shiny, contrasting denser, coarser punctation of mesoscutellum; smaller bees, head width < 1.6 mm	**Nannotrigona (Lispotrigona) Gonzalez & Engel**
36(34)	Bristle-like setae of vertex, mesoscutum, and mesoscutellum long, distinctly longer than median ocellar diameter	**37**
–	Bristle-like setae of vertex, mesoscutum, and mesoscutellum short, distinctly shorter than median ocellar diameter	**Scaptotrigona (Sakagamilla) Moure**
37(36)	Scape and supraclypeal area without minute, erect to suberect, bristle-like setae, at most sometimes with 1 or 2 suberect setae at extreme base of scape, otherwise setation minute and appressed; tergal setation not as below; integumental coloration variable	**38**
–	Scape along its length and supraclypeal area with numerous, minute, erect to suberect, bristle-like setae; all metasomal terga with dense, long, fine, erect, simple, yellow setae intermixed with similar short, appressed to decumbent setae; integument wholly yellow orange to orange	**Scaptotrigona (Dasytrigona) Engel**
38(37)	Discs of metasomal terga III–V with abundant, prominent, erect to subdecumbent, bristle-like setae, such setae frequently, but not universally, arising amid dense tomentum	**39**
–	Discs of metasomal terga III–V without bristle-like setae, instead with only fine, short to minute setae, such setae typically appressed to decumbent, if bristle-like setae present, then short (< 1/2 ocellar diameter) and confined to lateral margins or rarely sparse over disc and not associated with tomentum	**41**
39(38)	Metasomal terga III–V not covered in yellow tomentum, at most with diffuse areas of whitish or yellowish tomentum laterally on discs of terga IV–VI [care should be taken as sometimes the tomentum is difficult to see or may be largely rubbed off and only present in small lateral areas or under the margin of the preceding tergum]	**40**
–	Metasomal terga III–V covered with dense, yellow, plumose tomentum, typically obscuring integument [except in *Scaptotrigonafaviziae* Engel tomentum interrupted broadly medially, and largely absent on tergum III]	**Scaptotrigona (Scaptotrigona) Moure**
40(39)	Face below tangent of antennal toruli with a large yellow to yellowish brown patch, clypeus not concolorous with frons; upper frons with minute punctures well spaced, separated by 1–2× a puncture width	**Scaptotrigona (Baryorygma) Engel**
–	Face below tangent of antennal toruli brown to dark brown, largely concolorous with remainder of head, clypeus brown or concolorous with frons; upper frons with minute punctures dense, separated by much less than puncture width, nearly contiguous in places	**Scaptotrigona (Eoscaptotrigona) Engel**
41(38)	Metasomal terga III–V finely imbricate, somewhat shiny, with scattered punctures; mesoscutellum short, broadly rounded apically, apex extending only to basal margin of propodeum, not or barely overhanging propodeum	**Scaptotrigona (Astegotrigona) Engel**
–	Metasomal terga III–V coarsely imbricate to densely punctate; mesoscutellum long, apex somewhat blunt medio-apically, apex extending well past basal margin of propodeum and overhanging propodeum	**Scaptotrigona (Gymnotrigona) Engel**
42(33)	Mandible of worker with four apical teeth (lower two sometimes united by translucent septum but teeth still recognizable); mesoscutellum, as seen in lateral view projecting posteriorly as thin shelf over median part of metanotum [genus *Paratrigona* Schwarz, s.l.]	**43**
–	Mandible of worker with (rarely without) 1 or 2 denticles at upper end of apical margin, otherwise without teeth; mesoscutellum, as seen in lateral view, rather thick and rounded, not projecting as thin shelf over metanotum	**44**
43(42)	Metasomal terga shiny, in stark contrast with dull imbricate or coriaceous integument of head and mesosoma; setae quite conspicuous	**Paratrigona (Aparatrigona) Moure**
–	Metasomal terga dull imbricate or coriaceous, as on head and mesosoma; head, mesosoma, and terga typically with only exceedingly short and inconspicuous setae, rarely with more distinctly erect setae	**Paratrigona (Paratrigona) Schwarz**
44(42)	Metatibia of worker greatly broadened, spoon-shaped, ~ 3× as wide as metafemur, prolateral surface largely occupied by corbicula, proventral margin of metatibia with distal one-half convex; basal area of propodeum densely setose [genus *Partamona* Schwarz, s.l.]	**45**
–	Metatibia of worker not greatly broadened, < 3× as wide as metafemur, corbicula extending but little if at all basad middle of metatibia, proventral margin of metatibia convex only in distal 1/4 or less; basal area of propodeum usually asetose	**46**
45(44)	Cuticle of mesosoma shiny with minute, widely separated punctures; yellow of face pale and inconspicuous; metasomal terga without yellow maculations; worker gonostylus a rounded tubercle with few setae	**Partamona (Partamona) Schwarz**
–	Cuticle of mesosoma dull and minutely roughened; paraocular areas largely bright yellow; metasomal terga usually with yellow bands or lateral spots; worker gonostylus ~ 1.5× as long as broad, and setose	**Partamona (Parapartamona) Schwarz**
46(44)	Malar space much < 1/5 as long as compound eye; retrodorsal margin of metabasitarsus gently convex; yellow markings almost always present, at least on face	**47**
–	Malar space ~ 1/5 as long as compound eye; retrodorsal margin of metabasitarsus strongly convex medially; yellow markings absent	***Meliwillea* Roubik, Lobo Segura, & Camargo**
47(46)	Superior margin of retrolateral surface of metatibia not depressed, although shiny and in contrast to keirotrichiate area; concave surface of corbicula occupying full width of distal 1/2 of metatibia	**48**
–	Superior margin of retrolateral surface of metatibia strongly depressed, shiny, in sharp contrast to keirotrichiate area [except not depressed in apical 1/3 of metatibia of Scaura (Schwarzula)]; concave surface of corbicula usually not occupying whole distal 1/2 of metatibia	**49**
48(47)	Integument of head and mesosoma dull, microreticulate; preoccipital carina lamellate across upper part of head, with row of coarse setae, branched apically; supraclypeal area expanded laterally, forming flange partly covering antennal torulus; long setae lacking centrally on corbicular surface	***Paratrigonoides* Camargo & Roubik**
–	Integument largely shiny; preoccipital carina absent, without row of course setae; supraclypeal area not expanded laterally; 2 or 3 long setae present centrally on corbicular surface	***Nogueirapis* Moure**
49(47)	Metabasitarsus thickened, prolateral surface swollen, nearly as broad as or broader than metatibia, setae of inferior retrolateral margin curved apically; yellow maculation absent; rastellum nearly occupying full length of metatibial apical margin [genus *Scaura* Schwarz, s.l.]	**50**
–	Metabasitarsus not thickened, prolateral surface flat, much narrower than metatibia; setae of inferior retrolateral margin straight; yellow maculation present; rastellum occupying two-thirds or less of metatibial apical margin	**52**
50(49)	Metabasitarsus as wide as or wider than metatibia; malar space shorter than flagellar diameter; gena in profile narrower than compound eye; mandible virtually edentate	**51**
–	Metabasitarsus narrower than metatibia; malar space as long as flagellar diameter; gena in profile broader than compound eye; mandible with two denticles	**Scaura (Schwarzula) Moure**
51(50)	Metasoma elongate, length ≥ 3× width; metasomal tergum VI with dark fuscous or black setae	**Scaura (Scaura) Schwarz**
–	Metasoma subtriangular, length 1.5× width; metasomal tergum VI with white setae	**Scaura (Scauracea) Engel**
52(49)	Head and mesosoma largely smooth and shiny between scattered small setiferous punctures, rarely granulose-microrugulose and more matte; mesepisternum with at least some simple erect setae among plumose setae	**53**
–	Head and mesosoma with large, dense punctation or integument matte and microreticulate with indistinct punctures; mesepisternum with erect setae always plumose or minutely branched	**55**
53(52)	Mesoscutum shiny and smooth, punctures small to minute and distinctly separated, often widely so [genus *Plebeia* Schwarz, s.l.]	**54**
–	Mesoscutum generally matte owing to finely microrugulose-granulose sculpture resulting from dense, coarse, shallow punctures	***Asperplebeia* Engel**
54(53)	Forewing 2Cu a faint nebulous trace, weakening apically and disappearing by wing margin; minute bees, 2–3.5 mm in length	**Plebeia (Nanoplebeia) Engel**
–	Forewing 2Cu terminating on wing margin as a dark brown to brown tubular vein or nebulous trace, no weaker at terminus than on remigium; small bees, typically ≥ 3.5 mm in length	**Plebeia (Plebeia) Schwarz**
55(52)	Head and mesosoma with conspicuous yellow maculation; erect sternal setae predominantly plumose; moderate-sized bees, body length > 5 mm	**56**
–	Head and mesosoma with exceptionally small areas of yellow maculation; erect sternal setae simple; smaller bees, body length < 4 mm	***Friesella* Moure**
56(55)	Integument without metallic reflections; mesoscutum matte, integument microreticulate, with punctures indistinct [genus *Schwarziana* Moure, s.l.]	**57**
–	Integument with weak metallic reflections; mesoscutum shiny, punctures distinct and dense	***Mourella* Schwarz**
57(56)	Malar space shorter than flagellar diameter; mesepisternal setae long, longer than protibial width, and dense, sometimes obscuring integument	**Schwarziana (Schwarziana) Moure**
–	Malar space as long as flagellar diameter; mesepisternal setae short, shorter than protibial width, and sparser	**Schwarziana (Chapadapis) Engel**

### ﻿Genus *Asperplebeia* Engel

This is a recently established genus for two species of minute stingless bees formerly included in *Plebeia* Schwarz and occurring from southern Mexico to Costa Rica. The species look like smaller species of *Plebeia* (e.g., subgenus Nanoplebeia Engel), at only ~ 3 mm length, but can be distinguished by the generally more matte integument and coarser sculpturing. Nesting biology has only been studied for *Asperplebeiatica* (Wille), which nests in tree cavities and, unlike the superficially similar *Plebeia*, builds brood clusters rather than combs, although species of *Nanoplebeia* also build brood clusters (Roubik 2021).

- *A.moureana* (Ayala)
- *A.tica* (Wille)

### ﻿Genus *Cephalotrigona* Schwarz

*Cephalotrigona* Schwarz includes modestly large bees (8–10 mm), which are generally dark brown to black with faint yellow marks. Noteworthy for the genus is the carinate to lamellate preoccipital ridge, the abundant facial punctation, the long legs, and spatula- or racket-shaped metatibiae with broad corbiculae and only simple setae on the retromarginal edge. Nests are built in tree hollows, and the entrances are a bee-sized hole on a short, rounded platform, built only with cerumen and dark solid materials. The honey is of good quality, and they usually store propolis in abundance. Colonies are large but they are not easy to manage in meliponiculture. This genus occurs from Colima and Jalisco, as well as in Tamaulipas, Mexico to Santa Catarina, Brazil and Missiones, Argentina. The most common species between Mexico and Colombia is *Cephalotrigonazexmeniae* (Cockerell), similar in appearance to Trigona (Koilotrigona) fulviventris Guérin-Méneville since both are black with a reddish orange metasoma but is larger, while the most common in South America is *C.capitata* (Smith). Currently, there are only keys for the species from Mexico and Central America (Ayala 1999) and a revision of the genus is needed.

- *C.capitata* (Smith)
- *C.eburneiventer* (Schwarz)
- *C.femorata* (Smith)
- *C.oaxacana* Ayala
- *C.zexmeniae* (Cockerell)

### ﻿Genus *Duckeola* Moure

The two species of *Duckeola* Moure are robust bees of 8–9 mm length, found in Brazil, Ecuador, French Guiana, and Colombia. Perhaps the most striking feature of *Duckeola* is the considerably depressed parocular area between the compound eyes and the vertex, which elevates the vertex noticeably. In addition, the metatibia is noticeably claviform and lacks plumose setae on the retromarginal surface, and the mesotibial spur is absent (Oliveira 2002). No key to species is available but the two forms are quite distinct. Brood cells are arranged in clusters, although the nesting biology remains to be studied in detail.

- *D.ghilianii* (Spinola)
- *D.pavani* (Moure)

### ﻿Genus *Friesella* Moure

This is a genus of tiny bees, 3 mm in length, which look much like *Plebeia*, but have a reticulated matte integument, conspicuous abundant whitish pubescence, particularly on the face, and almost no yellow maculation on the face. The bees occur in southern Brazil and build irregular combs without an involucrum.

- *F.schrottkyi* (Friese)

### ﻿Genus *Frieseomelitta* Ihering

The genus *Frieseomelitta* Ihering includes slender species of 4–7 mm in length, ranging from Sinaloa and Veracruz, Mexico to Mato Grosso do Sul, Brazil. Recognition of the genus is aided by the presence of yellow marks on the face bordering the compound eyes on the paraocular area and genae, the absence of the mesotibial spur, the enlarged and inflated metatibia with a small corbicular depression restricted to the apical third, and an overall spatulate or racket-shaped to claviform metatibia with plumose setae on the retromarginal edge. The shape of the metasoma is subtriangular when constricted, in dorsal view, in the *varia* species group, while the metasoma is elongate, even when contracted, owing to broader terga II and III (this is the situation in the *nigra* and *portoi* species groups). The *nigra* species group has a claviform metatibia, while the *portoi* species group has a baseball-bat-shaped metatibia. Adults soon turn black on the head and mesosoma after emergence but the metasoma remains whitish for more than a week. The milky white wing tip in most species is another distinctive feature. The nests are distinctive for the arrangement of brood in clusters. New species are being described and a key to species developed by FFO (pers. obs.).

- *F.dispar* (Moure)
- *F.doederleini* (Friese)
- *F.flavicornis* (Fabricius)
- *F.languida* Moure
- *F.lehmanni* (Friese)
- *F.longipes* (Smith)
- *F.meadewaldoi* (Cockerell)
- *F.nigra* (Cresson)
- *F.paranigra* (Schwarz)
- *F.paupera* (Provancher)
- *F.portoi* (Friese)
- *F.silvestrii* (Friese)
- *F.trichocerata* Moure
- *F.varia* (Lepeletier)

### ﻿Genus *Geotrigona* Moure

As the name of the genus suggests, species of *Geotrigona* nest in cavities in the ground. The species are generally robust, 5–7 mm in length, with a short broad metasoma. The genus is distributed from Michoacan, in the central Balsas River depression in Mexico to Santiago de Estero, Argentina. Camargo and Moure (1996) and Gonzalez and Engel (2012) provided keys to the species.

#### SubgenusChthonotrigona Engel

- *G.acapulconis* (Strand)
- *G.chiriquiensis* (Schwarz)
- *G.fulvohirta* (Friese)
- *G.fumipennis* Camargo & Moure
- *G.joearroyoi* Gonzalez & Engel
- *G.kaba* Gonzalez & Sepúlveda
- *G.leucogastra* (Cockerell)
- *G.lutzi* Camargo & Moure
- *G.terricola* Camargo & Moure

#### SubgenusGeotrigona Moure, s.str.

- *G.aequinoctialis* (Ducke)
- *G.argentina* Camargo & Moure
- *G.fulvatra* Camargo & Moure
- *G.kraussi* (Schwarz)
- *G.kwyrakai* Camargo & Moure
- *G.mattogrossensis* (Ducke)
- *G.mombuca* (Smith)
- *G.subfulva* Camargo & Moure
- *G.subgrisea* (Cockerell)
- *G.subnigra* (Schwarz)
- *G.subterranea* (Friese)
- *G.tellurica* Camargo & Moure
- *G.xanthopoda* Camargo & Moure

### ﻿Genus *Lestrimelitta* Friese

This is the most diverse genus of robber stingless bees, occurring from Nayarit and San Luis Potosi, Mexico to Argentina. Species of *Lestrimelitta* Friese are cleptobiotic (Roubik 1980; Bego et al. 1991), maintaining their nests by “stealing” the resources of other Meliponini and introduced *Apis*, often causing losses to stingless beekeepers. *Lestrimelitta* primarily rob brood provisions from their hosts, most frequently species of *Nannotrigona*, *Scaptotrigona*, *Plebeia*, and *Melipona* (Sakagami et al. 1993). During an attack on the host bees’ nests, most *Lestrimelitta* release a pheromone reminiscent of lemon (citral), which confuses the defense communication between workers and guards of the host (Breed et al. 2004.). Keys to identification are presented by Camargo and Moure (1989), Oliveira and Marchi (2005), Marchi and Melo (2006), Gonzalez and Griswold (2012), and Guevara et al. (2020). The shape of the propodeal spiracle (Ayala 1999) and the dimensions of the mesotibial spur (Oliveira 2002) are two important characters that allow, together with the pattern of body setation, for the separation of species.

#### SubgenusApiraptor Engel

- *L.nana* Melo

#### SubgenusHyrolestris Engel

- *L.catira* Gonzalez & Griswold
- *L.chamelensis* Ayala
- *L.danuncia* Oliveira & Marchi
- *L.diminuta* Guevara, Gonzalez, & Ospina
- *L.ehrhardti* (Friese)
- *L.galvisi* Guevara, Gonzalez, & Ospina
- *L.glaberrima* Oliveira & Marchi
- *L.glabrata* Camargo & Moure
- *L.guyanensis* Roubik
- *L.huilensis* Gonzalez & Griswold
- *L.monodonta* Camargo & Moure
- *L.mourei* Oliveira & Marchi
- *L.niitkib* Ayala
- *L.rufipes* (Friese)
- *L.similis* Marchi & Melo

#### SubgenusLestrimelitta Friese, s.str.

- *L.chacoana* Roig-Alsina
- *L.ciliata* Marchi & Melo
- *L.limao* (Smith)
- *L.maracaia* Marchi & Melo
- *L.opita* Gonzalez & Griswold
- *L.piedemontana* Gonzalez & Rasmussen
- *L.rufa* (Friese)
- *L.spinosa* Marchi & Melo
- *L.sulina* Marchi & Melo
- *L.tropica* Marchi & Melo

### ﻿Genus *Melipona* Illiger

*Melipona* includes almost all of the most massive meliponines. These robust bees, 9–14 mm in length, with abundant plumose pubescence on the mesoscutum have some superficial resemblance to the largest African *Meliponula*. The wings are usually short and only reach the posterior end of the metasoma or slightly exceed it. The integument is generally black, but in some species brown or pale brown, with yellow, ivory, or brown areas, while the mesosoma has pale, brown, or dark pubescence, particularly the mesoscutum. The genus can be found from Sinaloa and south of Tamaulipas in Mexico to northern Argentina. Many species are used in meliponiculture, and these were the first ‘semi-domesticated’ bees, subject to multiplication and husbandry in the prehispanic Mayan culture. Nest products such as cerumen and honey have been extensively used through the ages. *Melipona* is now the largest genus in the tribe, but modern keys are lacking. A key to the species was provided by Schwarz (1932) and for Mexican species by Ayala (1999), with a recent reassessment of species by Camargo and Pedro (2007).

#### SubgenusEomelipona Moure

- *M.amazonica* Schulz
- *M.asilvai* Moure
- *M.bicolor* Lepeletier
- *M.carioca* Moure
- *M.carrikeri* Cockerell
- *M.marginata* Lepeletier
- *M.obscurior* Moure
- *M.ogilviei* Schwarz
- *M.picadensis* Strand
- *M.puncticollis* Friese
- *M.schencki* Gribodo
- *M.schwarzi* Moure
- *M.torrida* Friese
- *M.tumupasae* Schwarz

#### SubgenusMelikerria Moure

- *M.ambigua* Roubik & Camargo
- *M.beecheii* Bennett
- *M.compressipes* (Fabricius)
- *M.fasciculata* Smith
- *M.grandis* Guérin-Méneville
- *M.insularis* Roubik & Camargo
- *M.interrupta* Latreille
- *M.quinquefasciata* Lepeletier
- *M.salti* Schwarz
- *M.triplaridis* Cockerell

#### SubgenusMelipona Illiger, s.str.

- *M.baeri* Vachal
- *M.favosa* (Fabricius)
- *M.lunulata* Friese
- *M.lupitae* Ayala
- *M.mandacaia* Smith
- *M.orbignyi* (Guérin-Méneville)
- *M.peruviana* Friese
- *M.phenax* Cockerell
- *M.quadrifasciata* Lepeletier
- *M.subnitida* Ducke
- *M.variegatipes* Gribodo
- *M.yucatanica* Camargo, Moure, & Roubik

#### SubgenusMeliponiella Melo

- *M.bradleyi* Schwarz
- *M.illustris* Schwarz
- *M.micheneri* Schwarz

#### SubgenusMichmelia Moure

- *M.belizeae* Schwarz
- *M.boliviana* Schwarz
- *M.brachychaeta* Moure
- *M.capixaba* Moure & Camargo
- *M.captiosa* Moure
- *M.colimana* Ayala
- *M.costaricensis* Cockerell
- *M.cramptoni* Cockerell
- *M.crinita* Moure & Kerr
- *M.dubia* Moure & Kerr
- *M.eburnea* Friese
- *M.fasciata* Latreille
- *M.flavolineata* Friese
- *M.fulva* Lepeletier
- *M.fuscopilosa* Moure & Kerr
- *M.illota* Cockerell
- *M.indecisa* Cockerell
- *M.lateralis* Erichson
- *M.melanoventer* Schwarz
- *M.mimetica* Cockerell
- *M.mondury* Smith
- *M.nebulosa* Camargo
- *M.nigrescens* Friese
- *M.panamica* Cockerell
- *M.paraensis* Ducke
- *M.rufescens* Friese
- *M.rufiventris* Lepeletier
- *M.scutellaris* Latreille
- *M.seminigra* Friese
- *M.solani* Cockerell
- *M.trinitatis* Cockerell

#### SubgenusMouremelia Engel

- *M.fallax* Camargo & Pedro
- *M.fuliginosa* Lepeletier
- *M.titania* Gribodo

### ﻿Genus *Meliwillea* Roubik, Lobo Segura, & Camargo

This genus of little-known black bees from higher elevations (1400–2700 m) in Costa Rica and western Panama has an appearance superficially resembling *Partamona* Schwarz and *Scaptotrigona*. The shape of the metatibia is similar to that of *Scaptotrigona*, with a broader corbicula and with the characteristic large bristles arising from its surface as in *Partamona*.

- *M.bivea* Roubik, Lobo Segura, & Camargo

### ﻿Genus *Mourella* Schwarz

This is a monotypic genus, sister to the genus *Schwarziana* Moure, distributed in the southern portion of South America: Uruguay, Argentina, Paraguay, and Brazil (Paraná, Rio Grande do Sul, Santa Catarina). The sole species builds its nest in the soil and has architectural features typical to all other obligatory ground-nesting stingless bees (Camargo and Wittmann 1989). The head and mesosoma have conspicuous yellow maculation, while the remainder of the integument is weakly metallic, and the mesoscutum is largely shiny with distinct piligerous punctation. The genus is sometimes considered a distinctive subgenus at the base of *Schwarziana* (e.g., Engel et al. 2021a).

- *M.caerulea* (Friese)

### ﻿Genus *Nannotrigona* Cockerell

This genus includes small bees (4–4.5 mm) with largely black integument and areas of yellow maculation on the mesoscutum, mesoscutellum, and legs. The punctation of the head and mesosoma is quite coarse. The mesoscutellum is projected posteriorly over the metanotum and, like the genus *Scaptotrigona*, there is a polished mediolongitudinal depression on the anterior margin and a prominent, deep, U- or V-shaped notch in the apical margin medially. The genus extends from Sonora (Rio Mayo, Sonora), Mexico — the farthest north in Mexico for any stingless bee lineage — to the south of Brazil (Rio Grande do Sul). The bees nest in holes of all kinds, including those in the ground as well as in trees. Despite storing comparatively little honey, some species show potential for agricultural pollination and are easy to manage in meliponiculture for this purpose. The genus was revised by Rasmussen and Gonzalez (2017), supplemented by Jaramillo et al. (2019).

#### SubgenusLispotrigona Gonzalez & Engel

- *N.dutrae* (Friese)
- *N.schultzei* (Friese)

#### SubgenusNannotrigona Cockerell, s.str.

- *N.camargoi* Rasmussen & Gonzalez
- *N.chapadana* (Schwarz)
- *N.gaboi* Jaramillo, Ospina, & Gonzalez
- *N.melanocera* (Schwarz)
- *N.mellaria* (Smith)
- *N.minuta* (Lepeletier)
- *N.occidentalis* Jaramillo, Ospina, & Gonzalez
- *N.perilampoides* (Cresson)
- *N.pilosa* Jaramillo, Ospina, & Gonzalez
- *N.punctata* (Smith)
- *N.testaceicornis* (Lepeletier)
- *N.tristella* Cockerell

### ﻿Genus *Nogueirapis* Moure

*Nogueirapis* Moure differs from the closely related *Partamona* in the presence of abundant yellow markings; only slightly spoon-shaped, not greatly enlarged metatibia of the worker; as well as the smaller body size (3.5–5.5 mm), superficially resembling species of *Plebeia*. Like *Partamona*, there are one or two elongate bristles arising from the corbicular surface. Species nest in ground cavities but some also occasionally nest in tree hollows. A key to the species was presented by Ayala and Engel (2014) and Nogueira et al. (2020).

- *N.batistai* Nogueira
- *N.butteli* (Friese)
- *N.costaricana* Ayala & Engel
- *N.minor* (Moure & Camargo)
- *N.mirandula* (Cockerell)
- *N.rosariae* Nogueira

### ﻿Genus *Oxytrigona* Cockerell

This genus includes the infamous “fire bees”, so named because of their characteristic defense system. Workers have well-developed mandibular glands that produce a secretion containing formic acid, and which can inflict significant burns to the recipient of an attack. The bees are orange to black in color, with the integument of the head quite smooth and polished, sometimes with a vitreous appearance. Overall, the bees are typically 5–6 mm in length, with the head large, wider in comparison to the mesosoma, with an enlarged malar space and an interocular distance greater than the length of the compound eye itself. Species range from Chiapas, Mexico to southern Brazil (Santa Catarina). A key to the species was provided by Gonzalez and Roubik (2008).

- *O.chocoana* Gonzalez & Roubik
- *O.daemoniaca* Camargo
- *O.flaveola* (Friese)
- *O.huaoranii* Gonzalez & Roubik
- *O.ignis* Camargo
- *O.isthmina* Gonzalez & Roubik
- *O.mediorufa* (Cockerell)
- *O.mellicolor* (Packard)
- *O.mulfordi* (Schwarz)
- *O.obscura* (Friese)
- *O.tataira* (Smith)

### ﻿Genus *Paratrigona* Schwarz

Species of *Paratrigona* Schwarz are small, between 3.5–5.5 mm in length, and are generally black with well-delimited yellow markings. The mesoscutellum projects posteriorly over the metanotum as a plate, and the metasoma is typically robust, almost as wide as the mesosoma. The genus occurs from Veracruz, Mexico to northern Argentina (Salta), and southern Brazil (Rio Grande do Sul). Some species nest on the ground, while others build nests in various substrates, including wood and termite mounds and often on vines and within epiphytic plants. A key to the species was provided by Camargo and Moure (1994).

#### SubgenusAparatrigona Moure

- *P.impunctata* (Ducke)
- *P.isopterophila* (Schwarz)

#### SubgenusParatrigona Schwarz, s.str.

- *P.anduzei* (Schwarz)
- *P.catabolonota* Camargo & Moure
- *P.compsa* Camargo & Moure
- *P.crassicornis* Camargo & Moure
- *P.eutaeniata* Camargo & Moure
- *P.euxanthospila* Camargo & Moure
- *P.femoralis* Camargo & Moure
- *P.glabella* Camargo & Moure
- *P.guatemalensis* (Schwarz)
- *P.guigliae* Moure
- *P.haeckeli* (Friese)
- *P.incerta* Camargo & Moure
- *P.intermedia* Oliveira, Madella-Auricchio, & Freitas
- *P.lineata* (Lepeletier)
- *P.lineatifrons* (Schwarz)
- *P.lophocoryphe* Moure
- *P.lundelli* (Schwarz)
- *P.melanaspis* Camargo & Moure
- *P.myrmecophila* Moure
- *P.nuda* (Schwarz)
- *P.onorei* Camargo & Moure
- *P.opaca* (Cockerell)
- *P.ornaticeps* (Schwarz)
- *P.pacifica* (Schwarz)
- *P.pannosa* Moure
- *P.peltata* (Spinola)
- *P.permixta* Camargo & Moure
- *P.prosopiformis* (Gribodo)
- *P.rinconi* Camargo & Moure
- *P.scapisetosa* Gonzalez & Griswold
- *P.subnuda* Moure
- *P.uwa* Gonzalez & Vélez
- *P.wasbaueri* Gonzalez & Griswold

### ﻿Genus *Paratrigonoides* Camargo & Roubik

This genus superficially resembles *Paratrigona* or *Plebeia* with a dull integument, but has the keirotrichiate area of the metatibia not depressed on the superior margin; has yellow markings on the paraocular area, frontal median line, and spots below the lateral ocelli; and the upper part of the preoccipital ridge lamellate and bordered by a row of robust setae. The genus includes a single species of ~ 4.7 mm in length and is currently known only from Colombia. The nesting biology remains to be documented.

- *P.mayri* Camargo & Roubik

### ﻿Genus *Partamona* Schwarz

Species of *Partamona* are usually 5–6.5 mm in length, with a smooth and shiny integument, which can be black to orange-yellow, depending on the species, with vitreous yellow paraocular markings. The metatibia is quite large and broadened, making it distinctively spoon-shaped, and lacks plumose setae on the retromarginal edge. Species build semi-exposed nests on either natural or human constructions or on trees, as well as in the ground. Nest entrances are built of a material similar to hardened mud, often with a wide entrance. *Partamona* are not appropriate for meliponiculture as they are quite defensive and challenging to work with, in addition to insignificant amounts of stored honey. The genus occurs from Sonora, Mexico to southern Brazil (Rio Grande Do Sul). The species with testaceous bodies were revised by Camargo (1980), and the entire genus by Pedro and Camargo (2003).

#### SubgenusParapartamona Schwarz

- *P.brevipilosa* (Schwarz)
- *P.zonata* (Smith)

#### SubgenusPartamona Schwarz, s.str.

- *P.aequatoriana* Camargo
- *P.ailyae* Camargo
- *P.auripennis* Pedro & Camargo
- *P.batesi* Pedro & Camargo
- *P.bilineata* (Say)
- *P.chapadicola* Pedro & Camargo
- *P.combinata* Pedro & Camargo
- *P.criptica* Pedro & Camargo
- *P.cupira* (Smith)
- *P.epiphytophila* Pedro & Camargo
- *P.ferreirai* Pedro & Camargo
- *P.grandipennis* (Schwarz)
- *P.gregaria* Pedro & Camargo
- *P.helleri* (Friese)
- *P.littoralis* Pedro & Camargo
- *P.mourei* Camargo
- *P.mulata* Moure
- *P.musarum* (Cockerell)
- *P.nhambiquara* Pedro & Camargo
- *P.nigrior* (Cockerell)
- *P.orizabaensis* (Strand)
- *P.pearsoni* (Schwarz)
- *P.peckolti* (Friese)
- *P.rustica* Pedro & Camargo
- *P.seridoensis* Pedro & Camargo
- *P.sooretamae* Pedro & Camargo
- *P.subtilis* Pedro & Camargo
- *P.testacea* (Klug)
- *P.vicina* Camargo
- *P.vitae* Pedro & Camargo
- *P.xanthogastra* Pedro & Camargo
- *P.yungarum* Pedro & Camargo

### ﻿Genus *Plebeia* Schwarz

The genus *Plebeia* is a diverse group of often small and medium size bees (2–7 mm), with shiny integument bees and prominent yellow or white maculation. The metatibia is triangular shaped with only simple setae on the retromarginal edge, and the keirotrichiate zone of the retrolateral surface extends to the superior margin (without a shiny depressed rim). Included herein is the former genus Plectoplebeia Melo, an apparent synonym of the large subgenus Plebeia s.str. and representing merely larger, higher-elevation, seemingly cloud-forest specialized species of the subgenus (Engel 2022a). All features of *Plectoplebeia* intergrade into typical *Plebeia* s.str. as exemplified by comparison across species from one extreme to the other [e.g., *Plebeianigrifacies* (Friese), *P.aurantia* (Engel), *P.plectoforma* Engel, *P.hyperplastica* Engel, and *P.tigris* Engel] (Melo 2016; Engel 2022a). A key to the species occurring in Mexico and Central America was provided by Ayala (1999) and for Argentina by Alvarez et al. (2016). Species mostly nest in tree cavities and build brood combs. Two species nest exclusively in internodes in *Cecropia* Loefl. (Urticaceae), made available by ants, and interact with obligate inquiline scale insects that provide wax and honeydew (Roubik 2021, 2023). A key for the subgenus Nanoplebeia was provided by Engel (2021c).

#### SubgenusNanoplebeia Engel

- *P.asthenes* Engel
- *P.chondra* Engel
- *P.franki* (Friese)
- *P.margaritae* Moure
- *P.minima* (Gribodo)
- *P.orphne* Engel
- *P.pleres* Engel

#### SubgenusPlebeia Schwarz, s.str.

- *P.alvarengai* Moure
- *P.amydra* Engel
- *P.aurantia* (Engel), comb. n.
- *P.catamarcensis* (Holmberg)
- *P.cora* Ayala
- *P.deceptrix* Engel
- *P.droryana* (Friese)
- *P.emerina* (Friese)
- *P.emerinoides* (Silvestri)
- *P.flavocincta* (Cockerell)
- *P.frontalis* (Friese)
- *P.fulvopilosa* Ayala
- *P.goeldiana* (Friese)
- *P.grapiuna* Melo & Costa
- *P.guazurary* Alvarez, Rasmussen, & Abrahamovich
- *P.hyperplastica* Engel
- *P.jatiformis* (Cockerell)
- *P.julianii* Moure
- *P.kerri* Moure
- *P.llorentei* Ayala
- *P.lucii* Moure
- *P.malaris* Moure
- *P.manantlensis* Ayala
- *P.mansita* Alvarez & Rasmussen
- *P.melanica* Ayala
- *P.meridionalis* (Ducke)
- *P.mexica* Ayala
- *P.molesta* (Puls)
- *P.mosquito* (Smith)
- *P.mutisi* Engel
- *P.nigriceps* (Friese)
- *P.nigrifacies* (Friese)
- *P.parkeri* Ayala
- *P.peruvicola* Moure
- *P.phrynostoma* Moure
- *P.plectoforma* Engel
- *P.poecilochroa* Moure & Camargo
- *P.pulchra* Ayala
- *P.remota* (Holmberg)
- *P.roubiki* Engel
- *P.saiqui* (Friese)
- *P.silveirai* Engel
- *P.tigris* Engel
- *P.tobagoensis* Melo
- *P.variicolor* (Ducke)
- *P.vidali* Engel
- *P.wittmanni* Moure & Camargo

### ﻿Genus *Ptilotrigona* Moure

This genus greatly resembles *Tetragona* Lepeletier & Audinet-Serville in the presence of yellow maculation on the face and the velvety setation of the gena, the presence of a mesotibial spur, and the plumose setae on the retromarginal edge of the metatibia but can be distinguished by the setose basal area to the propodeum, the calviform metatibia with the proximal third more plump, and the larger mandibular teeth. The bees are 7–9 mm in length and extend from Costa Rica, with a noteworthy gap through Panama, thence to Colombia, central Brazil, and Peru. A key for the species was presented by Camargo (1996) and Camargo and Pedro (2004).

#### SubgenusCamargoia Moure

- *P.camargoi* (Moure)
- *P.nordestina* (Camargo)
- *P.pilicornis* (Ducke)

#### SubgenusPtilotrigona Moure, s.str.

- *P.lurida* (Smith)
- *P.occidentalis* (Schulz)
- *P.pereneae* (Schwarz)

### ﻿Genus *Scaptotrigona* Moure

*Scaptotrigona* is a distinctive group of small to medium-sized bees, 4.5–9 mm, which range in color from orange to black and lack yellow markings except for those on the postgena or elsewhere in one species of the subgenus Sakagamilla Moure [*Scaptotrigonaaffabra* (Moure)]. The preoccipital ridge is carinate to minutely lamellate and with three distinctive pits dorsally, and often with an interruption laterally. The metatibiae are also quite characteristic, subtriangular in shape and lacking plumose setae on the retromarginal edge, but bordered by an abundance of rather thick, curved bristles. Like *Nannotrigona* Cockerell, the mesoscutellum has a characteristic longitudinal depression or groove extending from anterior margin medially, but the integument is not as coarsely punctured. While the genus is easy to distinguish, the species are quite complex with considerable variation and the identification of the species can be difficult. The genus was recently organized into a series of subgenera, including a key to these subgeneric lineages (Engel 2022d). A key to species of the subgenus Sakagamilla is presented in Engel (2022b); to those of *Eoscaptotrigona* Engel, *Gymnotrigona* Engel, and *Baryorygma* Engel in Engel (2022d); to the species of *Astegotrigona* Engel by Engel (2022e). A partial key to one group of *Scaptotrigona* s.str. has been published (Engel 2022c) and other new species presented by Engel (2022f), but further work remains to be completed on the Central American fauna (in prep.) as well as *Scaptotrigona* s.str. in South America. The genus occurs from central Mexico to northern Argentina and is frequently found in meliponiaries. The nest entrance is a long trumpet or tube, sometimes greatly expanded, and usually with small uniformly spaced holes and much flexibility. Species nest almost solely in living trees (except when they live in buildings) and always build regular horizontal combs. A notable exception is *S.subobscuripennis* (Schwarz), of Costa Rica and western Panama, which nests in cavities in the ground, and apparently never uses tree cavities. The honey has good flavor and is appreciated by many, and propolis from *Scaptotrigona* is widely used for medicinal purposes. All species have pungent defensive cephalic secretions characterized as 2-nonal and are quite defensive.

#### SubgenusAstegotrigona Engel

- *S.ascheri* Engel
- *S.mexicana* (Guérin-Méneville)
- *S.wheeleri* (Cockerell)

#### SubgenusBaryorygma Engel

- *S.bipunctata* (Lepeletier)
- *S.emersoni* (Schwarz)
- *S.fimbriata* Engel
- *S.subobscuripennis* (Schwarz)
- *S.tricolorata* Camargo

#### SubgenusDasytrigona Engel

- *S.fulvicutis* Moure

#### SubgenusEoscaptotrigona Engel

- *S.luteipennis* (Friese)
- *S.polysticta* Moure
- *S.totobi* Engel

#### SubgenusGymnotrigona Engel

- *S.aurantipes* Engel
- *S.depilis* (Moure)
- *S.guimaraesensis* Laroca & Almeida
- *S.hellwegeri* (Friese)
- *S.jujuyensis* (Schrottky)
- *S.nuda* Engel
- *S.psile* Engel
- *S.stipula* Engel

#### SubgenusSakagamilla Moure

- *S.affabra* (Moure)
- *S.marialiceae* Laroca & Almeida
- *S.pasiphaea* Engel
- *S.silviae* Engel ^*^
- *S.tubiba* (Smith) ^*^

#### SubgenusScaptotrigona Moure, s.str.

- *S.anaulax* Engel
- *S.baldwini* Engel
- *S.barrocoloradensis* (Schwarz)
- *S.caduceus* Engel
- *S.ederi* Engel
- *S.extranea* Engel
- *S.faviziae* Engel
- *S.gonzalezi* Engel
- *S.grueteri* Engel
- *S.illescasi* Engel
- *S.kuperi* Engel
- *S.limae* (Brèthes), sp. inq.
- *S.macarenensis* Engel
- *S.magdalenae* Engel
- *S.nigrohirta* Nogueira & Santos-Silva
- *S.ochrotricha* (Buysson)
- *S.pectoralis* (Dalla Torre) [including *panamensis* (Cockerell)]
- *S.postica* (Latreille)
- *S.rosellae* Engel
- *S.santiago* Engel
- *S.semiflava* Engel
- *S.tatacoensis* Engel
- *S.turusiri* (Janvier), sp. inq.
- *S.vitorum* Engel
- *S.xanthotricha* Moure
- *S.yungasensis* Engel

### ﻿Genus *Scaura* Schwarz

This is a genus of small, 4–6 mm long, bees with slightly opaque black integument, without yellow markings, and superficially resemble darker species of *Plebeia*. The metabasitarsi are large and dilated, wider than the corresponding metatibiae and are used for rubbing floral structures to mop up loosened and scattered pollen. Nests are in tree cavities or even within the arboreal nests of nasutitermitine termites, and the bees build brood combs, except for *S.latitarsis* (Friese) and the species of *Schwarzula* Moure who build brood clusters and *S.longula* (Lepeletier) that builds simple vertical, double-sided appressed cell combs (Oliveira et al. 2013; Nogueira et al. 2023). The species of the subgenus Schwarzula further depart from this biology in that they tend scale insects that have an obligate nesting association and share wax and honeydew (such an association also exists independently in two species of *Nanoplebeia*: Roubik 2021, 2023). Information on the two species of this subgenus is provided by (Camargo and Pedro 2002a). A key to the species of *Scaura* s.str. and *Scauracea* Engel is provided by Nogueira et al. (2019). The genus is found from Veracruz, Mexico to southern Brazil and Bolivia.

#### SubgenusScaura Schwarz, s.str.

- *S.latitarsis* (Friese)
- *S.longula* (Lepeletier)

#### SubgenusScauracea Engel

- *S.amazonica* Nogueira, Oliveira, & Oliveira
- *S.argyrea* (Cockerell)
- *S.aspera* Nogueira & Oliveira
- *S.atlantica* Melo
- *S.cearensis* Nogueira, Santos Júnior, & Oliveira

#### SubgenusSchwarzula Moure

- *S.coccidophila* (Camargo & Pedro)
- *S.timida* (Silvestri)

### ﻿Genus *Schwarziana* Moure

This genus includes medium-sized bees, 6–8 mm in length, which were at one time placed among *Plebeia*. A key to species for the subgenus Schwarziana was provided by Melo (2015), at that time including subgenus Chapadapis Engel. Where known, the bees build nests in the ground, with the brood combs arranged in spirals. The genus is found in central-southern Brazil, and in Paraguay and northern Argentina.

#### SubgenusChapadapis Engel

- *S.chapadensis* Melo

#### SubgenusSchwarziana Moure, s.str.

- *S.bocainensis* Melo
- *S.mourei* Melo
- *S.quadripunctata* (Lepeletier)

### ﻿Genus *Tetragona* Lepeletier & Audinet-Serville

The bees of the genus *Tetragona* superficially resemble species of *Frieseomelitta*, as both include relatively long-legged bees, differing mainly by the presence of the mesotibial spur, the velvety pubescence of the gena, the yellow maculation of the head in most species (not extending to the top of the compound eyes and absent on the genae; *Tetragonaessequiboensis* (Schwarz) lacks yellow maculation entirely and species of the *handlirschii* group only have reddish yellow-brown areas on the clypeus), and the shape of the metatibiae, which are narrower. Species of *Tetragona* are ~ 5–8 mm in length and occur from Tabasco, Mexico to Uruguay. Species are used in meliponiculture and for supplies of sticky resin materials stored in nests. A key is provided by Nogueira et al. (2022) to species of the *clavipes* species group.

- *T.atahualpa* Nogueira & Rasmussen
- *T.beebei* (Schwarz)
- *T.clavipes* (Fabricius)
- *T.dorsalis* (Smith)
- *T.essequiboensis* (Schwarz)
- *T.goettei* (Friese)
- *T.handlirschii* (Friese)
- *T.kaieteurensis* (Schwarz)
- *T.korotaii* Nogueira
- *T.mayarum* (Cockerell)
- *T.mourei* Nogueira
- *T.perangulata* (Cockerell)
- *T.quadrangula* (Lepeletier)
- *T.truncata* Moure
- *T.ziegleri* (Friese)

### ﻿Genus *Tetragonisca* Moure

Species of *Tetragonisca* Moure are small (4–5 mm), slender bees, with yellow maculation on the lower portion of the face in most species, plumose setae on the retromarginal edge of the metatibia, and have a mesotibial spur (resembling *Tetragona*). However, the species of *Tetragonisca* have a basal sericeous area (an oval area with matted keirotrichia) on the retrolateral surface of the metabasitarsus of workers and lack velvety pilosity on the gena. The metatibia is also rather inflated, with the corbicula reduced to the apical portion of the podite. The genus extends from Veracruz, Mexico to northern Argentina. It should be noted that the widespread and common *Tetragoniscaangustula* (Latreille) appears to be a complex of species. The systematics of this species should be explored in depth. Nests are built in tree cavities or the ground. No key to species is currently available.

- *T.angustula* (Latreille)
- *T.buchwaldi* (Friese)
- *T.fiebrigi* (Schwarz)
- *T.weyrauchi* (Schwarz)

### ﻿Genus *Trichotrigona* Camargo & Moure

This is a genus of enigmatic stingless bees from northern Brazil. The genus includes bees of 5–6 mm in length, and that superficially resemble *Frieseomelitta* but have conspicuous setae on the compound eyes (hence the generic name). Species of *Trichotrigona* Camargo & Moure are likely robber bees but seemingly undertaking isolated, rather than mass, raids. Pedro and Cordeiro (2015) provide a means to distinguish the two species.

- *T.camargoiana* Pedro & Cordeiro
- *T.extranea* Camargo & Moure

### ﻿Genus *Trigona* Jurine

This is a genus with bees of dramatically different proportions, ranging in size from 5–12 mm in length, with plumose setae on the retromarginal edge of the metatibia, velvety pilosity on the gena, a mesotibial spur (resembling *Tetragona*), and can be reddish orange to black in coloration, but always lack yellow maculation. The mandibles have well-developed, prominent teeth (either four or five). The metatibiae are claviform and in one subgenus (*Necrotrigona* Engel) the corbicula is not developed. Aside from the raised median keirotrichiate area on the retrolateral surface of the metatibia, there is also a basal sericeous area (an oval area with matted keirotrichia) on retrolateral surface of the metabasitarsus. The biology of *Trigona* is remarkably varied, perhaps more so than any other genus of Meliponini, and includes obligate necrophages that scavange from carcasses (*Necrotrigona*). Species nest in squirrel or bird nests as well as in tree cavities, while some nest on the ground among tree roots and others build exposed nests around tree branches. The largest nests are composed of the bees’ own feces, from defecated pollen exines. This group now includes 30 described species but will likely be found to have more than twice as many, like *Trigonisca* (Rasmussen and Camargo 2008). The subgenus Nostotrigona was revised recently, and a key was provided to the species (Ribeiro et al. 2023).

#### SubgenusAphaneura Gray

- *T.chanchamayoensis* Schwarz
- *T.ferricauda* Cockerell
- *T.muzoensis* Schwarz
- *T.pallens* (Fabricius)
- *T.williana* Friese

#### SubgenusAphaneuropsis Engel

- *T.cilipes* (Fabricius)
- *T.lacteipennis* Friese
- *T.mazucatoi* Almeida
- *T.pellucida* Cockerell

#### SubgenusDichrotrigona Engel

- *T.dimidiata* Smith
- *T.sesquipedalis* Almeida
- *T.venezuelana* Schwarz

#### SubgenusKoilotrigona Engel

- *T.braueri* Friese
- *T.fulviventris* Guérin-Méneville
- *T.guianae* Cockerell

#### SubgenusKtinotrofia Engel

- *T.albipennis* Almeida
- *T.fuscipennis* Friese

#### SubgenusNecrotrigona Engel

- *T.crassipes* (Fabricius)
- *T.hypogea* Silvestri
- *T.necrophaga* Camargo & Roubik

#### SubgenusNostotrigona Engel

- *T.daianeae* Ribeiro
- *T.juvenili* Ribeiro
- *T.mandaloriana* Ribeiro
- *T.permodica* Almeida
- *T.recursa* Smith

#### SubgenusTrigona Jurine, s.str.

- *T.amalthea* (Olivier)
- *T.amazonensis* (Ducke)
- *T.branneri* Cockerell
- *T.corvina* Cockerell
- *T.dallatorreana* Friese
- *T.hyalinata* (Lepeletier)
- *T.nigerrima* Cresson
- *T.pampana* Strand
- *T.silvestriana* (Vachal)
- *T.spinipes* (Fabricius)
- *T.truculenta* Almeida

### ﻿Genus *Trigonisca* Moure

The genus *Trigonisca* includes exclusively minute stingless bee species and is the earliest-diverging lineage of extant Neotropical Meliponini. The genus is one of the more widespread groups of New World stingless bees, as well as a relatively commonly encountered group of meliponines. The nesting biology was explored for *Trigoniscamepecheu* Engel & Gonzalez by Engel et al. (2019). A key to the species of *Celetrigona* Moure is presented by Camargo and Pedro (2009). The subgenera *Leurotrigona* and *Exochotrigona* Engel apparently form a grade at the base of *Trigonisca* s.l. and are classified by some authors as a separate genus. They can be easily distinguished from the other Neotropical minute bees by the smooth, polished, and shiny integument, the absence of a preoccipital carina, and the absence of yellow markings. Many new species have been encountered and are currently being described for the western Amazon (Roubik 2018). A key to the species was provided by Pedro and Camargo (2009: all species at that time classified in *Leurotrigona*). A revision of *Trigonisca* s.str. is needed, but a beginning was made by Albuquerque and Camargo (2007). Camargo and Pedro (2005) provided a key to species for *Dolichotrigona*, although this group almost assuredly renders *Trigonisca* s.str. paraphyletic and is therefore not recognized herein. Nonetheless, for readers who prefer to recognize *Dolichotrigona* we provide the following couplet that distinguishes those species from *Trigonisca* s.strictiss.

**Table:** 

1	Integument densely and strongly micro-alveolate; metatibia long and narrow, > 3× longer than wide; scape and flagellomeres I–III longer than wide, somewhat compressed	**Trigonisca (Dolichotrigona) Moure**
–	Integument more faintly microveolate, ranging from slightly matte to faintly glossy; metatibia shorter and wider, < 3× longer than wide; scape and flagellomeres I–III shorter and less compressed	**Trigonisca (Trigonisca) Moure, *s.strictiss*.**

#### SubgenusCeletrigona Moure

- *T.euclydiana* (Camargo & Pedro)
- *T.hirsuticornis* (Camargo & Pedro)
- *T.longicornis* (Friese)
- *T.manauara* (Camargo & Pedro)

#### SubgenusExochotrigona Engel

- *T.crispula* (Pedro & Camargo)
- *T.pusilla* (Moure & Camargo)

#### SubgenusLeurotrigona Moure

- *T.gracilis* (Pedro & Camargo)
- *T.muelleri* (Friese)

#### SubgenusTrigonisca Moure, s.str.

- *T.atomaria* (Cockerell)
- *T.azteca* Ayala
- *T.bidentata* Albuquerque & Camargo
- *T.browni* (Camargo & Pedro) [*Dolichotrigona*]
- *T.buyssoni* (Friese)
- *T.chachapoya* (Camargo & Pedro) [*Dolichotrigona*]
- *T.clavicornis* (Camargo & Pedro) [*Dolichotrigona*]
- *T.coephloei* (Schwarz)
- *T.discolor* (Wille)
- *T.dobzhanskyi* (Moure)
- *T.duckei* (Friese)
- *T.extrema* Albuquerque & Camargo
- *T.flavicans* (Moure)
- *T.fraissei* (Friese)
- *T.graeffei* (Friese)
- *T.hirticornis* Albuquerque & Camargo
- *T.intermedia* Moure
- *T.longitarsis* (Ducke) [*Dolichotrigona*]
- *T.martinezi* (Brèthes) [*Dolichotrigona*]
- *T.maya* Ayala
- *T.mendersoni* (Camargo & Pedro) [*Dolichotrigona*]
- *T.mepecheu* Engel & Gonzalez
- *T.meridionalis* Albuquerque & Camargo
- *T.mixteca* Ayala
- *T.moratoi* (Camargo & Pedro) [*Dolichotrigona*]
- *T.nataliae* (Moure)
- *T.pediculana* (Fabricius)
- *T.pipioli* Ayala
- *T.rondoni* (Camargo & Pedro) [*Dolichotrigona*]
- *T.roubiki* Albuquerque & Camargo
- *T.sachamiski* Alvarez & Lucia
- *T.schulthessi* (Friese) [*Dolichotrigona*]
- *T.tavaresi* (Camargo & Pedro) [*Dolichotrigona*]
- *T.townsendi* (Cockerell)
- *T.unidentata* Albuquerque & Camargo
- *T.variegatifrons* Albuquerque & Camargo
- *T.vitrifrons* Albuquerque & Camargo

### ﻿Afrotropical Meliponini

The fauna of stingless bees in Africa and Madagascar is the least diverse of any in the world. Afrotropical Meliponini are unique and found in a line from Senegal to Eritrea along the southern Sahel southward to KwaZulu-Natal, South Africa. The tribe is also found throughout Madagascar. Currently we recognize 33 extant species in the Afrotropical fauna and eight extant genera (Table 2).

**Table 2.** Hierarchical classification of Eastern Hemisphere stingless bees (Meliponini: Hypotrigonina).

Subtribe **Hypotrigonina** Engel
[Old World Meliponini]
Infratribe Heterotrigonitae Engel
*Heterotrigona* Genus Group
Genus *Geniotrigona* Moure
Genus *Heterotrigona* Schwarz, s.l.
Subgenus Borneotrigona Engel
Subgenus Sundatrigona Inoue & Sakagami
Subgenus Heterotrigona Schwarz, s.str.
Subgenus Platytrigona Moure
Subgenus Sahulotrigona Engel & Rasmussen
Genus *Papuatrigona* Michener & Sakagami
Genus *Lepidotrigona* Schwarz
Genus *Wallacetrigona* Engel & Rasmussen
*Homotrigona* Genus Group
Genus *Homotrigona* Moure, s.l.
Subgenus Lophotrigona Moure
Subgenus Homotrigona Moure, s.str.
Subgenus Odontotrigona Moure
Subgenus Tetrigona Moure
*Tetragonula* Genus Group
Genus *Tetragonula* Moure, s.l.
Subgenus Tetragonilla Moure
Subgenus Tetragonula Moure, s.str.
Infratribe Hypotrigonitae Engel
*Hypotrigona* Genus Group
Genus *Hypotrigona* Cockerell
Genus *Liotrigona* Moure, s.l.
Subgenus Cleptotrigona Moure
Subgenus Liotrigona Moure, s.str.
Subgenus †*Tapheiotrigona* Engel
Genus *Pariotrigona* Moure
Genus *Lisotrigona* Moure
Genus *Ebaiotrigona* Engel & Nguyen
Genus *Austroplebeia* Moure, s.l.
Subgenus †*Anteplebeina* Engel
Subgenus Austroplebeia Moure, s.str.
Genus †*Kelneriapis* Sakagami
Genus †*Liotrigonopsis* Engel
*Meliponula* Genus Group
Genus *Meliplebeia* Moure, s.l.
Subgenus Apotrigona Moure
Subgenus Meliplebeia Moure, s.str.
Genus *Axestotrigona* Moure, s.l.
Subgenus Atrichotrigona Engel
Subgenus Axestotrigona Moure, s.str.
Genus *Plebeiella* Moure
Genus *Dactylurina* Cockerell
Genus *Meliponula* Cockerell
Genus *Plebeina* Moure
Genus †*Adactylurina* Engel
Subtribe *Incertae sedis*
Genus †*Meliponorytes* Tosi [Hypotrigonitae?]

### ﻿Key to genera and subgenera of African Meliponini (modified from Engel et al. 2021a)

**Table:** 

1	Forewing length < 3.5 mm; hind wing without closed cells, veins closing radial and cubital cells, if visible at all, clear and unpigmented; forewing with 2Rs, 1rs-m, and 2rs-m almost always completely absent, thus indications of submarginal cells absent; at least distal part of second cubital cell (= subdiscoidal cell) of forewing undefined or defined by completely unpigmented vein traces; vein 2M of forewing terminating without bend at about position of anterior end of 1m-cu (i.e., 3M absent), which, however, is absent or spectral (sensu Mason 1986; = barely discernible)	**2**
–	Forewing length ~ 4 mm or more; hind wing commonly with radial and cubital cells closed by at least weakly brownish nebulous veins; forewing with 2Rs and 1rs-m usually weakly indicated, first submarginal cell thus usually recognizable; second cubital cell of forewing completely indicated, at least by faint veins; vein M of forewing extending at least slightly beyond position of anterior end of 1m-cu and angulate at end of that crossvein (i.e., 3M distinct from 2M, with at least tubular to nebulous stub), which is usually at least faintly visible	**4**
2(1)	Distal part of prolateral surface of metatibia flat or concave, bordered by long setae, forming corbicula; penicillum present; clypeus twice as wide as long or less	**3**
–	Prolateral surface of metatibia convex, without corbicula; penicillum absent; clypeus much > 2× as wide as long	**Liotrigona (Cleptotrigona) Moure**
3(2)	Superior distal angle of metatibia forming distinct angle; mesoscutum and mesoscutellum smooth and shiny; gonostyli much longer than broad, flat, adjacent, or separated by less than one gonostylar width, without setae but with gonotrichia	**Liotrigona (Liotrigona) Moure**
–	Superior distal angle of metatibia a rounded contour; mesoscutum and mesoscutellum matte, micro-alveolate to imbricate; gonostyli minute, tuberculiform, separated by several gonostylar widths, with setae but without gonotrichia	***Hypotrigona* Moure**
4(1)	Retrolateral surface of metatibia with depressed upper marginal glabrate area narrow (much < 1/2 as wide as broad area with keirotrichia) or absent, keirotrichia extending to or close to margin; first metasomal segment broader than long	**5**
–	Retrolateral surface of metatibia with strongly depressed, shiny, superior marginal glabrate area nearly as broad apically as longitudinal median keirotrichiate plateau, and ~ 1/2 as wide as keirotrichiate plateau midway of metatibial length; first metasomal segment longer than broad	***Dactylurina* Cockerell**
5(4)	Metatibia rather spoon-shaped, superior distal angle rounded but with coarse, amber-colored to blackish setae (superior parapenicillum); sting stylet distinct, acute	**6**
–	Metatibia slender, triangular with distinct superior distal angle supporting long, pale setae (not especially coarse); sting stylet a mere rounded convexity	***Plebeina* Moure**
6(5)	Propodeal profile with slanting dorsal portion rounding onto vertical portion; corbicula occupying more than distal half of metatibia; metasomal terga at least partly shiny [apical reflexed process of sternum VI of male longer than body of sternum]	**7**
–	Propodeal profile largely vertical; corbicula occupying less than distal half of metatibia; metasomal terga dull, minutely sculptured [apical reflexed process of sternum VI of male short and rounded]	***Meliponula* Cockerell**
7(6)	Head and mesosoma without yellow markings; retrolateral surface of metatibia without well-defined, shiny, depressed superior margin, although keirotrichiate not reaching margin, at least distally [genus *Axestotrigona* Moure, s.l.]	**8**
–	Head and mesosoma with yellow markings; retrolateral surface of metatibia with shiny superior margin, at least slightly depressed	**9**
8(7)	Basal area of propodeum finely tessellate to microalveoate, sometimes faintly so and appearing nearly smooth, and laterally setose (sometimes lateral patches of setae sparse and wispy or difficult to discern in worn or dirty individuals, such as with considerable resin on body); wing membranes hyaline clear to lightly infuscate (parchment-colored) or ferruginous	**Axestotrigona (Axestotrigona) Moure**
–	Basal area of propodeum glabrous and smooth; wing membranes darkly infumate throughout	**Axestotrigona (Atrichotrigona) Engel**
9(7)	Basal area of propodeum pubescent [genus *Meliplebeia* Moure, s.l.]	**10**
–	Basal area of propodeum glabrous	***Plebeiella* Moure**
10(9)	Mesoscutum tessellate; mandibular teeth small; scape as long as antennal-ocellar distance; basal vein (1M) slightly distad 1cu-a; superior parapenicillum well developed	**Meliplebeia (Meliplebeia) Moure**
–	Mesoscutum punctate; mandibular teeth strong; scape shorter than antennal-ocellar distance; basal vein (1M) slightly basad 1cu-a; superior parapenicillum scarcely definable	**Meliplebeia (Apotrigona) Moure**

### ﻿Genus *Axestotrigona* Moure

*Axestotrigona* Moure is a genus of modest-sized bees, 5–7 mm in length, and lacking yellow integumental markings. The keirotrichiate zone of the metatibial retrolateral surface extends all the way to retromarginal edge, and therefore is easily distinguished from the genera *Meliplebeia* Moure and *Plebeiella* Moure. The brood cells are arranged in horizontal combs and the nests are are built within pre-existing cavities in trees or in the sides of earthen termite nests. A key to the species is provided by Solórzano-Kraemer et al. (2022).

#### SubgenusAtrichotrigona Engel

- *A.cameroonensis* (Friese)
- *A.simpsoni* Moure

#### SubgenusAxestotrigona Moure, s.str.

- *A.erythra* (Schletterer)
- *A.ferruginea* (Lepeletier)
- † *A.kitingae* Engel & Solórzano-Kraemer
- *A.togoensis* (Stadelmann)

### ﻿Genus *Dactylurina* Cockerell

*Dactylurina* Cockerell is perhaps the most distinctive genus of African stingless bees. As the name implies, the metasoma is elongate, thin, and subclavate, giving it a finger-like shape. The genus occurs from Guinea eastward to the Congo and Uganda and thence southward to Angola. The genus is distinctive for building double vertical combs (e.g., Michener 1974; Njoya et al. 2016), much like *Apis* Linnaeus, and also shared by the Neotropical *Scaura* s.str. (Nogueira et al. 2023).

- *D.schmidti* (Stadelmann)
- *D.staudingeri* (Gribodo)

### ﻿Genus *Hypotrigona* Cockerell

Like *Liotrigona* Moure, *Hypotrigona* Cockerell includes minute stingless bees commonly encountered in Africa from Senegal eastward to Eritrea, and southward to northern South Africa. The genus is distinctive for the broadly rounded distal superior angle of the metatibia and the dull, matte, reticulate to micro-alveolate integument. The genus is also commonly encountered in East African copal and modern resins (Solórzano-Kraemer et al. 2022). Nest may be built in dry logs, rock or wall crevices, or even in pre-existing cavities of living trees, often with a protruding oval entrance tube. The brood are arranged in clusters or irregular layers (e.g., Portugal-Araújo 1955; Michener 1959; Kajobe 2007; Ndungu et al. 2019).

- *H.araujoi* (Michener)
- *H.gribodoi* (Magretti)
- † *H.kleineri* Engel & Solórzano-Kraemer
- *H.ruspolii* (Magretti)
- *H.squamuligera* (Benoist)

### ﻿Genus *Liotrigona* Cockerell

This is a genus of minute stingless bees and occurs commonly throughout Madagascar as well as less predominantly on the African continental mainland from Liberia eastward to Ethiopia and southward to the northern half of South Africa. The species can be confused with *Hypotrigona* but differ in the smooth and shiny integument and the presence of a distinct superior distal angle on the metatibia. The monotypic subgenus Cleptotrigona Moure includes a species that is a robber bee on *Hypotrigona* and other *Liotrigona*. A single extinct species is also known from Early Miocene amber from Ethiopia (Engel and Aber 2022). Like most minute stingless bees, the brood cells are clustered rather than arranged in distinct combs (e.g., Brooks and Michener 1988; Hora et al. 2023).

#### SubgenusCleptotrigona Moure

- *L.cubiceps* (Friese)

#### SubgenusLiotrigona Moure, s.str.

- *L.baleensis* Pauly & Hora
- *L.betsimisaraka* Pauly
- *L.bitika* Brooks & Michener
- *L.bottegoi* (Magretti)
- *L.bouyssoui* (Vachal)
- *L.chromensis* Pauly
- *L.gabonensis* Pauly & Fabre Anguilet
- *L.kinzelbachi* Koch
- *L.madecassa* (Saussure)
- *L.mahafalya* Brooks & Michener
- *L.nilssoni* Michener
- *L.parvula* (Darchen)
- † *L.vetula* Moure & Camargo
- *L.voeltzkovi* (Friese)

#### Subgenus †*Tapheiotrigona* Engel

- † *L.aethiopica* Engel

### ﻿Genus *Meliplebeia* Moure

*Meliplebeia* includes species superficially resembling the smaller *Plebeiella* and found from Gambia to Eritrea and Somaliland, and southward to Namibia and northern South Africa. Unlike *Plebeiella*, the basal area of the propodeum is pubescent. Nests are built like those described for *Axestotrigona (supra)*.

#### SubgenusApotrigona Moure

- *M.nebulata* (Smith)

#### SubgenusMeliplebeia Moure, s.str.

- *M.beccarii* (Gribodo)
- *M.gambiana* Moure
- *M.roubiki* (Eardley)

### ﻿Genus *Meliponula* Cockerell

The genus *Meliponula* includes a modestly large and robust species, 6–8 mm in length, found commonly throughout tropical Africa, from Guinea eastward to Kenya and thence southward to Namibia and Botswana. The genus is distinctive for the wholly declivitous propodeal basal area, dull and matte metasomal terga, and restriction of the corbicula to less than the distal half of the metatibia. Nests are constructed within pre-existing cavities in trees, and the brood are arranged in irregular layers (Portugal-Araújo 1955) or can also be within ground cavities. There is a pattern for nests in the highlands to always be belowground, while those of lower elevations are in tree hollows (e.g., Kajobe 2007).

- *M.bocandei* (Spinola)

### ﻿Genus *Plebeiella* Moure

The genus *Plebeiella* includes small bees most similar to *Meliplebeia* but differing in the glabrous basal area to the propodeum. The genus occurs from Togo eastward to Kenya and southward to Angola and Zambia. Nests are built like those described for *Axestotrigona (supra)*.

- *P.griswoldorum* (Eardley)
- *P.lendliana* (Friese)

### ﻿Genus *Plebeina* Moure

This genus superficially resembles the New World *Plebeia* and includes a small species (4–5 mm in length) that can be found from Senegal eastward to Ethiopia and then southward to Angola and northeastern South Africa. Nests are built like those described for *Axestotrigona (supra)*.

- *P.armata* (Magretti)

### ﻿Indomalayan, Papuasian, and Australian Meliponini

The fauna of stingless bees across South and Southeast Asia, through the Indomalayan and Papuasian regions, and into Australia is the richest in the Eastern Hemisphere, with a particularly interesting diversity extending across Indomalaya and Papuasia. A catalogue of the fauna was provided by Rasmussen (2008). All meliponines from these regions belong to the subtribe Hypotrigonina (Table 2), a group which also includes the African lineages as well as likely encompasses those species preserved in European and Asian amber sources (Engel et al. 2021a). Currently we recognize 98 extant species in the fauna and 11 extant genera.

### ﻿Key to genera and subgenera of Indomalayan, Papuasian, and Australian Meliponini

Еxpanded from Rasmussen et al. 2017; Engel and Rasmussen 2017; Engel et al. 2018, 2022; Engel 2019.

**Table:** 

1	Forewing length < 3 mm, wing venation greatly reduced and retromargin of metatibia without plumose setae; hind wing without closed cells, veins closing radial and cubital cells, if visible at all, clear and unpigmented (spectral: sensu Mason 1986); forewing with 2Rs and 1rs-m almost always completely absent, thus without indication of submarginal cells; at least distal part of second cubital cell of forewing undefined or defined completely by unpigmented spectral vein traces (i.e., at least 2Cu and 3Cu absent or spectral); vein M of forewing terminating without bend (i.e., 3M lacking) at about position of anterior end of where 1m-cu (which is absent) would occur	**2**
–	Forewing length typically > 4 mm, wing venation typically not greatly reduced for Meliponini, but *if* minute and with some wing reduction, then retromargin of metatibia with plumose setae intermixed with simple setae; hind wing typically with radial and cubital cells closed by at least faintly brownish nebulous veins; forewing with one or two submarginal cells usually weakly indicated by nebulous traces of 2Rs and 1rs-m, first submarginal cell usually recognizable; second cubital cell of forewing completely indicated by at least faint nebulous veins (i.e., 2Cu present); vein M of forewing usually extending at least slightly beyond position of 1m-cu and angular at apex of tubular portion of vein (i.e., 3M present), the stub of which is usually at least faintly visible	**4**
2(1)	Malar space shorter than flagellar diameter; inner margins of compound eyes converging below	**3**
–	Malar space almost 1/5 as long as compound eye, much longer than flagellar diameter; inner margins of compound eyes nearly parallel	***Pariotrigona* Moure**
3(2)	Yellow maculation present in worker on scape, supraclypeal area, clypeus, pronotal lobe and sometimes on lower paraocular area, apically on mesoscutellum, and laterally on mesoscutum; scape without erect setae; minutely plumose facial setae absent on upper frons; gonocoxae unmodified, with gonostyli articulating more distally; gonostyli elongate, bladelike, expanded and lamellate proximally; genital capsule rectigonal; metasomal sternum VI medio-apically chamfered, bilobed	***Ebaiotrigona* Engel & Nguyen**
–	Yellow maculation lacking, at most with pale yellow brown areas; scape with erect setae; minutely plumose facial setae extending across upper frons; gonocoxae with enormous, arched, proximal extensions, with gonostyli articulating near midlength; gonostyli slender elongate; genital capsule schizogonal; metasomal sternum VI with a single medio-apical process	***Lisotrigona* Moure**
4(1)	Mesosoma and usually head without distinct maculation; retrolateral surface of metatibia with strong longitudinal keirotrichiate ridge above which is a broad, depressed, shiny marginal area	**5**
–	Mesoscutellum and usually face and mesoscutum with well-developed yellow maculation; retrolateral surface of metatibia with keirotrichiate area broad, nearly reaching retrodorsal margin of metatibia	***Austroplebeia* Moure**
5(4)	Retromarginal setae of worker metatibia and males entirely simple, or some plumose setae only on apical 1/5 or 1/6 of margin; keirotrichiate median zone of retrolateral surface of metatibia separated from shiny superior marginal subglabrate zone by gentle slope (gentle clivulus)	**6**
–	Retromarginal setae of worker metatibia and some males partly plumose; elevated keirotrichiate median zone of retrolateral surface of metatibia separated from shiny superior marginal subglabrate zone by abrupt slope (abrupt clivulus)	**7**
6(5)	Mesoscutum margined with whitish, densely plumose, scale-like setae; head and mesosoma dull, with minute close punctures; propodeal dorsum finely reticulate; retrodorsal margin of worker metatibia without plumose setae	***Lepidotrigona* Schwarz**
–	Mesoscutum without conspicuous plumose setae; head and mesosoma shiny, although with minute, rather close punctures; propodeal dorsum smooth, shiny; retrodorsal margin of worker metatibia with plumose setae among setae on apical 1/5 or 1/6 of margin	***Papuatrigona* Michener & Sakagami**
7(5)	Mesoscutellum well projected posteriorly, extending over propodeum as far as posterior propodeal angle (change in slope between basal area and posterior surface) (best seen in profile); malar area linear (= exceedingly narrow to virtually lacking with compound eye appearing to abutt mandibular articulations) or at least narrower than 0.5× diameter of flagellomere III; vein M of forewing straight and ending at or shortly after 1m-cu [genus *Tetragonula* Moure, s.l.]	**8**
–	Mesoscutellum short, only slightly projecting over metanotum (best seen in profile); malar area variable, typically as long as diameter of flagellomere III or greater but sometimes ~ 0.5–0.75× diameter of flagellomere III; vein M of forewing bent at trace of 1m-cu, sometimes present only as minute stub beyond bend	**9**
8(7)	Scape shorter than torulocellar distance; ~ 5 distal hamuli; retromarginal contour of metatibia slightly convex, with superior distal angle subangulate; rastellum and penicillum usually composed of soft setae; forewing membrane rather uniformly colored, typically clear to lightly infuscate; pleural setae pale; forewing marginal cell nearly closed, sometimes with apex of Rs bent and nebulous (i.e., appendiculate), with or without 2r-rs stub arising at bend	**Tetragonula (Tetragonula) Moure**
–	Scape at least as long as torulocellar distance; 6 distal hamuli; retromarginal contour of metatibia distinctly and broadly convex, with superior distal angle rounded, almost without angulation; rastellum and penicillum composed of stiff setae; forewing membrane markedly bicolored, proximally darkly fuscate; pleural setae fuscous to black; forewing marginal cell more broadly opened apically, apex of Rs never bent (i.e., never appendiculate)	**Tetragonula (Tetragonilla) Moure**
9(7)	Malar space < 2× diameter of flagellomere III	**10**
–	Malar space ≥ 2× diameter of flagellomere III	**18**
10(9)	Mandible unidentate or bidentate, teeth small [genus *Heterotrigona* Schwarz, s.l.]	**11**
–	Mandible bidentate, teeth large, deeply incised, i.e., interdental spaces deep [genus *Homotrigona* Moure, s.l.]	**15**
11(10)	Basal area of propodeum largely or entirely glabrous, at most with apicolateral patches of setae, if patches present, then broad glabrous area much wider than setal patches and occupying majority of propodeal basal surface	**12**
–	Basal area of propodeum entirely pubescent or with a narrow medial glabrous area, if glabrous area present, then distinctly narrower than lateral setose areas, frequently width approximately equivalent to medial length of metanotum	**13**
12(11)	Basal vein (1M) basad 1cu-a; wings strongly bicolorous, proximal portion (darkly infumate in costal, radial, and first cubital cells) contrasting with clear apical portion; mesoscutum and mesoscutellum with abundant, erect, thick, stiff, black, bristle-like setae (similar to those of *Heterotrigona* s.str.); superior marginal subglabrate zone of metatibial retrolateral surface apically broader than keirotrichiate zone	**Heterotrigona (Borneotrigona) Engel**
–	Basal vein (1M) confluent with or slightly distad 1cu-a; wings not bicolorous, proximal half generally similar in color to apical half; mesoscutum and mesoscutellum without such erect, thick, stiff, black setae (some species may have fuscous setae but never the thickened, stiff, bristle-like setae); superior marginal subglabrate zone of metatibial retrolateral surface apically narrower than keirotrichiate zone	**Heterotrigona (Platytrigona) Moure**
13(11)	Basal vein (1M) basad 1cu-a; basal area of propodeum glabrous, without small, wispy apicolateral patches of setae	**14**
–	Basal vein (1M) distad 1cu-a; basal area of propodeum largely glabrous but with small, wispy, apicolateral patches of setae	**Heterotrigona (Sahulotrigona) Engel & Rasmussen**
14(13)	Superior marginal subglabrate zone of metatibial retrolateral surface apically broader than keirotrichiate zone; larger bees, forewing length greater than 6 mm	**Heterotrigona (Heterotrigona) Schwarz**
–	Superior marginal subglabrate zone of metatibial retrolateral surface apically narrower than or at most as broad as keirotrichiate zone; smaller bees, forewing length < 6 mm	**Heterotrigona (Sundatrigona) Inoue & Sakagami**
15(10)	Basal sericeous area of metabasitarsus present; clypeus ~ 2× broader than long	**16**
–	Basal sericeous area of metabasitarsus absent; clypeus short, ≥ 2.5× broader than long	**Homotrigona (Homotrigona) Moure**
16(15)	Basal area of propodeum smooth and glabrous; vertex not elevated posterior to ocelli	**17**
–	Basal area of propodeum pubescent; vertex elevated posterior to ocelli	**Homotrigona (Lophotrigona) Moure**
17(16)	Malar space as long as flagellar diameter; clypeus with a transverse row of erect setae along apical margin; metabasitarsus 2× as long as wide	**Homotrigona (Tetrigona) Moure**
–	Malar space about as long as 1.5× flagellar diameter; clypeus with erect black setae scattered over entire surface; metabasitarsus < 1.5× as long as wide	**Homotrigona (Odontotrigona) Moure**
18(9)	Vertex with deep depression and elevated ridge rising above level of ocelli, posteriorly without deep, concave, medial notch; mesoscutum with dense covering of short, plumose setae amid scattered erect, black setae; apical metasomal terga with dense, long, apically plumose setae amid erect, black setae, with plumose setae at least as long as black setae; keirotrichiate zone of metatibial retrolateral surface narrower than superior subglabrate zone, and greater than length of apical subglabrate zone	***Geniotrigona* Moure**
–	Vertex without strongly elevated ridge, with faint transverse depression and ridge posterior to ocelli, posteriorly with deep, concave medial incision; mesoscutum without dense covering of short, plumose setae amid scattered erect, black setae; apical metasomal terga with short, scattered plumose setae amid longer, erect, black setae; keirotrichiate zone of metatibial retrolateral surface about as broad as or slightly broader than superior subglabrate zone, and subequal to length of apical subglabrate zone	***Wallacetrigona* Engel & Rasmussen**

### ﻿Genus *Austroplebeia* Moure

*Austroplebeia* Moure superficially resembles the African *Plebeina* Moure and the New World *Plebeia*, particularly in the presence of prominent yellow maculation on the head and mesosoma. The genus occurs in New Guinea southward through northern Australia. A key to the species was provided by Dollin et al. (2015), while a key to the two subgenera was presented by Engel et al. (2021a).

#### Subgenus †*Anteplebeina* Engel

- † *A.fujianica* Engel

#### SubgenusAustroplebeia Moure, s.str.

- *A.australis* (Friese)
- *A.cassiae* (Cockerell)
- *A.cincta* (Mocsáry)
- *A.essingtoni* (Cockerell)
- *A.magna* Dollin, Dollin, & Rasmussen

### ﻿Genus *Ebaiotrigona* Engel & Nguyen

The sole species of this genus of minute bees is found in Southeast Asia and was originally classified in *Liotrigona*. Recently, however, the discovery of the male demonstrated that the species was more dramatically different from true *Liotrigona* than originally surmised. Instead, the type species seems more similar to *Austroplebeia* and was therefore reclassified (Engel et al. 2022). Nests are built in rock crevices and the brood cells are built in irregular clusters (Engel et al. 2022).

- *E.carpenteri* (Engel)

### ﻿Genus *Geniotrigona* Moure

*Geniotrigona* Moure includes large robust stingless bees with a prominent elevated ridge on the vertex posterior to the ocelli, a long malar space, and dense plumose setae on the mesosoma. The genus occurs from Southeast Asia through Malesia. A key to the species was provided by Rasmussen et al. (2017).

- *G.lacteifasciata* (Cameron)
- *G.thoracica* (Smith)

### ﻿Genus *Heterotrigona* Schwarz

The genus *Heterotrigona* Schwarz includes species similar to *Homotrigona* Moure, but distinctly smaller in size and much reduced mandibular dentition. Three subgenera (*Borneotrigona* Engel, *Heterotrigona* s.str., and *Sundatrigona* Inoue & Sakagami) occur west of the Wallace Line, while the remaining two are found exclusively east of the line. Keys to the species of *Platytrigona* Moure and *Sahulotrigona* Engel & Rasmussen were provided by Engel (2019), while a key distinguishing the two species of *Sundatrigona* was provided by Sakagami et al. (1990).

#### SubgenusBorneotrigona Engel

- *H.hobbyi* (Schwarz)

#### SubgenusHeterotrigona Schwarz, s.str.

- *H.bakeri* (Cockerell)
- *H.erythrogastra* (Cameron)
- *H.itama* (Cockerell)

#### SubgenusPlatytrigona Moure

- *H.flaviventris* (Friese)
- *H.keyensis* (Friese)
- *H.lamingtonia* (Cockerell)
- *H.planifrons* (Smith)

#### SubgenusSahulotrigona Engel & Rasmussen

- *H.paradisaea* Engel & Rasmussen
- *H.taraxis* Engel
- *H.tricholoma* Engel

#### SubgenusSundatrigona Inoue & Sakagami

- *H.lieftincki* (Sakagami & Inoue)
- *H.moorei* (Schwarz)

### ﻿Genus *Homotrigona* Moure

*Homotrigona* includes those larger species with pronounced mandibular dentition. Some authors have afforded the individual subgenera generic rank (e.g., Moure 1961). To emphasize the close relationship of these groups we follow a more conservative approach whereby they are considered subgenera of a more inclusive and more readily circumscribed genus. The genus in this concept extends from Southeast Asia through Malesia, but not crossing the Weber Line.

#### SubgenusHomotrigona Moure, s.str.

- *H.aliceae* (Cockerell)
- *H.anamitica* (Friese)
- *H.fimbriata* (Smith)
- *H.lutea* (Bingham)

#### SubgenusLophotrigona Moure

- *H.canifrons* (Smith)

#### SubgenusOdontotrigona Moure

- *H.haematoptera* (Cockerell)

#### SubgenusTetrigona Moure

- *H.apicalis* (Smith)
- *H.binghami* (Schwarz)
- *H.melanoleuca* (Cockerell)
- *H.peninsularis* (Cockerell)
- *H.vidua* (Lepeletier)

### ﻿Genus *Lepidotrigona* Schwarz

As the generic name implies, the genus is noteworthy for the presence of abundant, short, decumbent, plumose (scale-like) setae covering the mesoscutal surface. Additionally, the integument is generally dull and matte, the basal area of the propodeum is reticulate, and the simple setae of the metatibial retromarginal edge. The metatibia and associated corbicular surface, of some species, is greatly expanded, resulting in a spoon-shaped leg, and often associated with an expanded metabasitarsus [e.g., *Lepidotrigonanitidiventris* (Smith), *L.palavanica* (Cockerell), *L.latipes* (Friese)]. Other species have a more typical metatibia with a smaller corbicular surface, such as *L.arcifera* (Cockerell), while there are those that have a seemingly intermediary form between the extremes (e.g., *L.satun* Attasopa & Bänziger). The genus occurs from India eastward to the Philippines and then across Malesia.

- *L.amruthae* Vikratamath & Thangjam
- *L.arcifera* (Cockerell)
- *L.doipaensis* (Schwarz)
- *L.flavibasis* (Cockerell)
- *L.hoozana* (Strand)
- *L.javanica* (Gribodo)
- *L.latebalteata* (Cameron)
- *L.latipes* (Friese)
- *L.nitidiventris* (Smith)
- *L.palavanica* (Cockerell)
- *L.rajithae* Vikratamath & Thangjam
- *L.satun* Attasopa & Bänziger
- *L.sikkimensis* Vikratamath
- *L.terminata* (Smith)
- *L.thenzawlensis* Vikratamath & Thangjam
- *L.trochanterica* (Cockerell)
- *L.ventralis* (Smith)

### ﻿Genus *Lisotrigona* Moure

This is a genus of minute, tear- and sweat-drinking stingless bees found across South and Southeast Asia (D.W. Roubik, *in litt*., found both species drinking sweat). A key to the species, at that time including *Ebaiotrigonacarpenteri* (Engel), was provided by Engel (2000b).

- *L.cacciae* (Nurse)
- *L.furva* Engel

### ﻿Genus *Papuatrigona* Michener & Sakagami

This is a monotypic genus of stingless bees endemic to New Guinea. The genus has some features reminiscent of the New World *Oxytrigona* Cockerell and it would be worth investigating whether or not *Papuatrigona* Michener & Sakagami is similarly aggressive with defensive compounds. The nesting biology remains to be studied in detail.

- *P.atricornis* (Smith)

### ﻿Genus *Pariotrigona* Moure

This is a genus of minute stingless bees from Southeast Asia and western Malesia. The species resembles *Lisotrigona* Moure and *Ebaiotrigona* Engel & Nguyen, from the same region, but has a long malar space and parallel compound eyes. *Ebaiotrigona* further differs from both in the presence of yellow facial maculation, and all three differ quite dramatically in the form of the male terminalia (Michener 2001, 2007b; Engel et al. 2022). Some authors have recognized two species. The bees nest on limestone outcrops and build elaborate coral-like nest entrances. Like the species of *Lisotrigona*, *Pariotrigona* Moure is lachryphagous (Bänziger et al. 2011).

- *P.pendleburyi* (Schwarz)

### ﻿Genus *Tetragonula* Moure

The genus *Tetragonula* Moure is the most diverse and widespread of all Old World stingless bees, extending from western India to central-eastern Australia. Many of the species can be exceedingly similar, differing in seemingly minor details. Simultaneously, individuals within any given species, even within a single nest, may also be quite variable in aspects of coloration and some proportions. There are regional keys to species (e.g., Sakagami 1978), but no comprehensive monograph and the identification of species in some areas of tropical Asia can be challenging given the absence of keys and modern evaluations of their circumscriptions. Ultimately, some synonymy may well be recognized, potentially older names may need to be resurrected from synonymy, and some new species may continue to be discovered. The genus is also quite old, with a species of subgenus Tetragonula s.str. preserved in mid-Miocene amber from Zhangpu, China (Engel et al. 2021a).

#### subgenus Tetragonilla Moure

- *T.atripes* (Smith)
- *T.collina* (Smith)
- *T.fuscibasis* (Cockerell)
- *T.rufibasalis* (Cockerell)

#### subgenus Tetragonula Moure, s.str.

- *T.ashishi* Viraktamath & Jagruti
- *T.bengalensis* (Cameron)
- *T.biroi* (Friese)
- *T.callophyllae* Shanas & Faseeh
- *T.carbonaria* (Smith)
- *T.clypearis* (Friese)
- *T.dapitanensis* (Cockerell)
- *T.davenporti* (Franck)
- *T.drescheri* (Schwarz)
- † *T.florilega* Engel
- *T.fuscobalteata* (Cameron)
- *T.geissleri* (Cockerell)
- *T.gressitti* (Sakagami)
- *T.hirashimai* (Sakagami)
- *T.hockingsi* (Cockerell)
- *T.iridipennis* (Smith)
- *T.kyrdemkulaiensis* Viraktamath & Thangjam
- *T.laeviceps* (Smith)
- *T.malaipanae* Engel, Michener, & Boontop
- *T.melanocephala* (Gribodo)
- *T.melina* (Gribodo)
- *T.mellipes* (Friese)
- *T.minangkabau* (Sakagami & Inoue)
- *T.minor* (Sakagami)
- *T.pagdeni* (Schwarz)
- *T.pagdeniformis* (Sakagami)
- *T.penangensis* (Cockerell)
- *T.perlucipinnae* Shanas & Faseeh
- *T.praeterita* (Walker)
- *T.reepeni* (Friese)
- *T.ruficornis* (Smith)
- *T.sapiens* (Cockerell)
- *T.sarawakensis* (Schwarz)
- *T.shishirae* Viraktamath
- *T.shubhami* Viraktamath
- *T.sirindhornae* (Michener & Boongird)
- *T.srikantanathi* Viraktamath
- *T.sumae* Viraktamath
- *T.testaceitarsis* (Cameron)
- *T.vikrami* Viraktamath
- *T.zucchii* (Sakagami)

### ﻿Genus *Wallacetrigona* Engel & Rasmussen

*Wallacetrigona* Engel & Rasmussen is endemic to mountainous areas of Sulawesi and was previously classified in *Geniotrigona*, but the species lacks the many specializations of the latter genus (see comment for *Geniotrigona*) while having a characteristic U-shaped incision in the posterior margin of the vertex, among other character combinations (Rasmussen et al. 2017). The bees nest in pre-existing tree cavities, with the brood arranged in horizontal combs (Suhri et al. 2022).

- *W.incisa* (Sakagami & Inoue)

### ﻿Extinct genera

The remaining genera are exclusively known from fossil species, all preserved in ambers ranging in age from the Late Cretaceous (Maastrichtian) to the early Miocene (Burdigalian). Extinct species in genera that are still living (e.g., *Tetragonulaflorilega* Engel) are listed above under their respective clades.

#### ﻿† Adactylurina

Taxon classificationAnimaliaHymenopteraApidae

Engel gen. nov.

CAC143B2-627D-5F85-AF40-B13A7ACA925D

https://zoobank.org/F0829A32-01BC-44C6-9F66-453E52DF900A

##### Type species.

*Dactylurinaaethiopica* Lepeco & Melo, 2022.

##### Diagnosis.

This species in Miocene amber from Ethiopia was originally placed in the genus *Dactylurina*. It differs quite notably from *Dactylurina* and is therefore here removed to a new genus. The fossil genus differs from *Dactylurina* in the absence of a basal sericeous area on the retrolateral surface of the metabasitarsus (such an area is present in *Dactylurina*), the metasoma that is roughly cylindrical and tapers apically (metasoma greatly elongate, finger-like, and subclavate in *Dactylurina*), face not wider than compound eye length (wider than compound eye length in *Dactylurina*), and two preapical teeth of the mandible (unidentate in *Dactylurina*).

##### Etymology.

The new genus-group name is a combination of the Ancient Greek alpha privative *a*– / *ᾰ̓*–, indicating negation, and *Dactylurina* Cockerell [itself a combination of the Latin adjective *dactylus*, meaning, “finger-like” (from Ancient Greek *dáktulos* / *δᾰ́κτῠλος*, meaning, “finger”), and the noun *ūrīna*, meaning, “urine” but also referring more generally to “genitals” or even metaphorically to the “tail end” through its Ancient Greek origins from the word *ourā́* / *οὐρᾱ́*, meaning, “tail”], the genus to which the species was originally placed. The gender of the name is feminine.

- † *A.aethiopica* (Lepeco & Melo), comb. nov.

### ﻿Genus †*Cretotrigona* Engel

This is an interesting fossil preserved in amber from New Jersey that dates from near the end of the Maastrichtian and is therefore the earliest fossil Meliponini and also the oldest definitive bee.

- † *C.prisca* (Michener & Grimaldi)

### ﻿Genus †*Exebotrigona* Engel & Michener

This genus was described for a *Trigonisca*-like species of Meliponini in Fushun amber, but subsequent studied indicated that the fossil was not in Eocene amber from China but instead likely from the Baltic region. The provenance of the holotype needs considerable study.

- † *E.velteni* Engel & Michener

### ﻿Genus †*Kelneriapis* Sakagami

*Kelneriapis* Sakagami is known only from a single worker preserved in Eocene Baltic amber. Engel (2001a) revised the genus and species. This is a minute stingless bee similar to the extant African genus *Liotrigona* and the extinct genus *Liotrigonopsis* Engel from the same deposits.

- † *K.eocenica* (Kelner-Pillault)

### ﻿Genus †*Liotrigonopsis* Engel

This genus, like the two preceding genera, is known only from middle Eocene Baltic amber. Currently, there is only a single worker known. The genus and species were characterized by Engel (2001a) and is a minute stingless bee similar in morphology to the extant African genus *Liotrigona*.

- † *L.rozeni* Engel

### ﻿Genus †*Meliponorytes* Tosi

This genus is known only from Miocene Sicilian amber. The original material has not been re-examined since the end of the 19^th^ century and so it remains a poorly understood group, but likely belongs to the Hypotrigonina (Rasmussen and Cameron 2010; Engel et al. 2021a), based on the original description and figures.

- † *M.sicula* Tosi
- † *M.succini* Tosi

### ﻿Genus †*Proplebeia* Michener

This is a genus of *Plebeia*-like bees occurring in middle Miocene amber of the Dominican Republic and southern Mexico.

- † *P.abdita* Greco & Engel
- † *P.dominicana* (Wille & Chandler)
- † *P.silacea* (Wille)
- † *P.tantilla* Camargo, Grimaldi, & Pedro
- † *P.vetusta* Camargo, Grimaldi, & Pedro

### ﻿Biology

Stingless bees are, of course, eusocial (Michener 1974), and of the anchored grade (sensu Engel 2023). They live in perennial colonies, which range considerably in size and complexity, but always have males and females, the latter organized into distinct worker and queen castes, and the usual division of labor, age polyethism, and generation overlaps that come from these distinctions (Fig. 6). In addition, polyethism within the worker caste, most often based on age, is sometimes also based on subtle morphometric differences to produce, in some species, a functional soldier-like caste (Grüter et al. 2017). Workers dominate the population of a colony and undertake all of the work to maintain the nest and support the queen and males. Most notably they perform all of the foraging activities for food and nest materials, and throughout the active period of the day a nearly constant flow of foraging workers can be seen coming and going. While for most stingless bees this consists of collecting pollen, nectar, resin, and mud, there are exceptions, with some species harvesting honeydew and wax from scale insects (Coccidae), collecting from fungi, and others scavenging the decomposing carcasses of vertebrates (Camargo and Pedro 2002b; Oliveira and Morato 2000; Roubik 1982, 2021). Scavenging the carcasses of small vertebrates occurs, but larger vertebrates are used by obligate necrophages, who also utilize invertebrates like annelids, spiders and Orthoptera. Several species of Meliponini and other bees visit carcasses to collect salts, lipids, and water (Oliveira et al. 2013, Ribeiro et al. 2022), but only obligate necrophages consume animal protein and make honey from dead meat, and microbes make the glucose and amino acids (Roubik 2023). In Asia, minute species, where known, are lachryphagous, sucking the tears from eyes of various birds and mammals (Bänziger 2018). In the Neotropical region lachryphagous species of *Trigonisca* s.l. (typically the subgenera *Leurotrigona*, *Exochotrigona*, and some *Trigonisca* s.str.), are known as “lambe-olhos”, “lameojos”, or “chupaojos” (lick eye) (Camargo and Pedro 2005; pers. obs.), and some species when crushed leave a caustic secretion that can cause eye irritation (Carvalho et al. 1949; Villas-Bôas and Villas-Bôas 1994). Foraging for pollen may even be decoupled from visiting flowers in *Scaura*, where the bees use enlarged metabasitarsi to “mop” pollen that has fallen from flowers onto bordering leaves or other surfaces (Michener et al. 1978). A similar behavior was observed for Trigonisca (Trigonisca) tavaresi Camargo & Pedro whereby the bees collected pollen from the surface of flower petals that had been dropped by larger bees (Oliveira et al. 2013). At an even greater extreme, species of *Lestrimelitta* and Liotrigona (Cleptotrigona) are robbers that collect their materials from the nests of other meliponines, either by mass invasion, as in the former or by stealthy, spy-like gathering by isolated workers in the latter (Portugal-Araújo 1958; Sakagami et al. 1993). *Trichotrigona* apparently also does not store food and is a cleptobiotic social bee associated with *Frieseomelitta* (Pedro and Cordeiro 2015).

From the moment of eclosion to the adult, workers begin their life-long labors, with lifespans ranging from 30–40 days (Sakagami 1982). Initially they are teneral, appearing nearly white and with comparatively soft cuticle (called callows), but gradually the tanning and melanization of sclerites is completed. For the first phase of their lives, workers remain within the colony to care for the developing young, building brood combs and storage pots, process food resources, engage in hygienic behaviors, cleaning and repairing the nest, tending the queen, thermal regulation for the nest, and, when necessary, defense from invaders, among many other roles (Grüter, 2020). Workers within the colony may also lay eggs, which are either trophic eggs used to feed the queen or develop into males given that workers are unmated and their eggs are therefore unfertilized (Koedam et al. 1996). As they age, workers take on the more dangerous roles external to the nest, primarily foraging. Males will also forage for nutrients as they do not receive food within the nest, nor do they live within the nest. Foraging workers locate, harvest, and transport the many supplies needed to keep colonial life going (van Veen and Sommeijer 2000; Sakagami 1982). Additionally, during colony division such foragers will locate a new suitable nest site and begin construction of the new nest, with the materials coming from nature or even derived from the original nest. Eventually, that subset of workers that established the new nest will be followed by an unmated queen that will then copulate with males and assume the reproductive role for the new colony. This is, of course, in stark contrast to honey bees (Apini) in which it is the old queen that departs to establish the new colony (Michener 2007a). Gradually, interaction and exchange between the original and new colonies cease, which results in two distinct and functional societies.

Most colonies have a single physogastric queen (Fig. 6A), although there are uncommon cases of polygynous colonies occurring naturally (e.g., Velthuis et al. 2001). Queens are produced in larger brood cells, queen cells (Fig. 6), which are built on the periphery of combs and typically formed by combining two normal brood cells (Grüter 2020). In *Melipona*, however, the brood cells are of a uniform size. In Meliponini queens are determined by diet and a genetic-feeding mechanism (Kerr 1948), but in *Melipona* and some species that build brood cells in clusters virtually any emerging female is capable of becoming a potential queen dependent on what she is fed by the earlier generation of workers. Unmated queens can be found in colonies at nearly any time of year and may even be imprisoned by the workers in entrapment cells composed of cerumen or even in empty storage pots in the case of *Melipona* (Imperatriz-Fonseca and Zucchi 1995). The small population of unmated queens provides the colony a source of replacement queens should the current queen die unexpectedly. In meliponiaries there is in *Melipona* the phenomenon of parasitic virgin queens, which enter nests lacking queens, but might or might not do so in native forest environments (Roubik 2023). When colonies are about to divide, the production of unmated queens will increase. If too may unmated queens are present, then workers may expel them from the nest, kill them outright, or imprison them. Either way, the result is that such expelled queens will die in the absence of a nest and support from a retinue of workers. Often a queen will store considerable nutrients within her body, resulting in the expansion of her metasoma. Nonetheless, once mated the queen’s metasoma will expand dramatically owing to the development of her ovaries and an increase in the number of ovarioles. Many mated queens will become so physogastric as to be unable to fly.

Males are usually the least common individuals within a nest. Males usually appear when the number of royal cells increases and there is a preponderance of food stores within the nest. The emerging males do little other than hang about the periphery of the nest or cluster around unmated queens, waiting for them to depart on their nuptial flight at which point they have a chance of mating. During times of scarcity, any males are often expelled and killed to avoid their wasting of resources.

### ﻿Nesting biology and architecture

**Figure 5.**
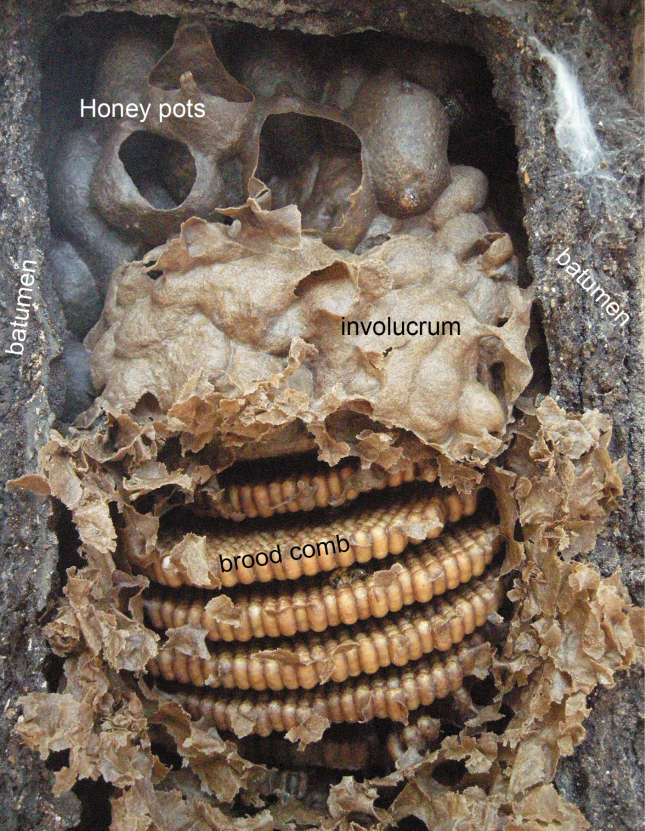
Cross-section of a nest of Melipona (Melikerria) beecheii Bennett from near Tapachula, Chiapas, Mexico, with key architectural elements labeled. Photograph M.A. Guzmán Díaz (used with permission).

**Figure 6.**
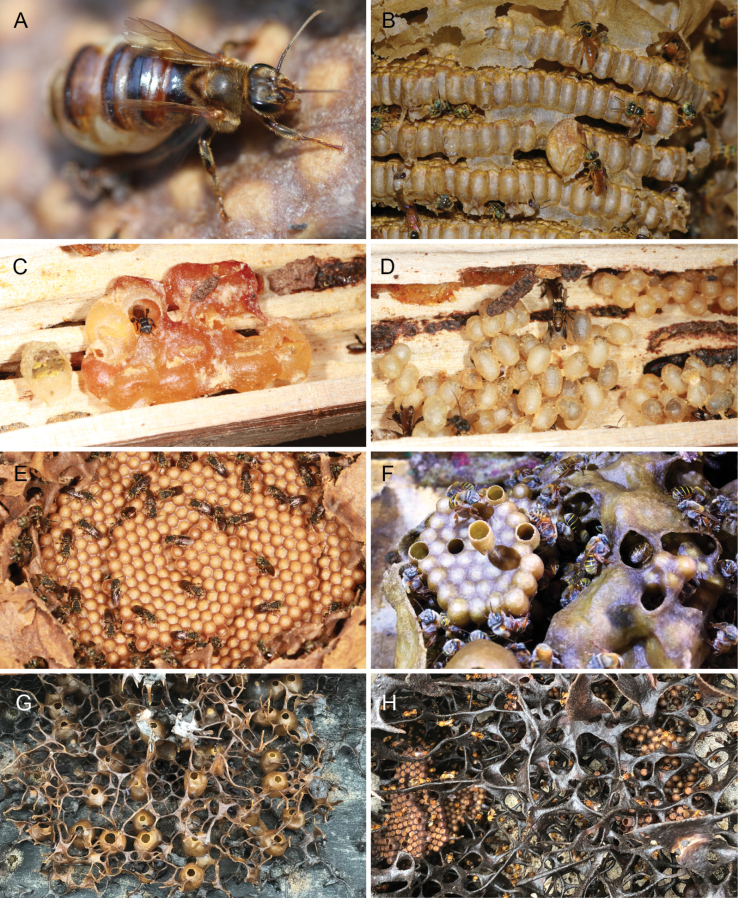
Nests of representative stingless bees **A** physogastric queen of Melipona (Michmelia) illota Cockerell from Peru **B** colony of *Tetragoniscaangustula* (Latreille) from Colombia **C, D** colony with honey pots of Scaura (Scaura) latitarsis (Friese) from Peru **E**Nannotrigona (Nannotrigona) melanocera (Schwarz) from Peru **F**Melipona (Melikerria) beecheii Bennett from Mexico **G**Heterotrigona (Heterotrigona) itama (Cockerell) from Brunei **H***Geniotrigonalacteifasciata* (Cameron) from Brunei. All photographs C. Rasmussen except F R. Ayala and G and H M.S. Engel.

**Figure 7.**
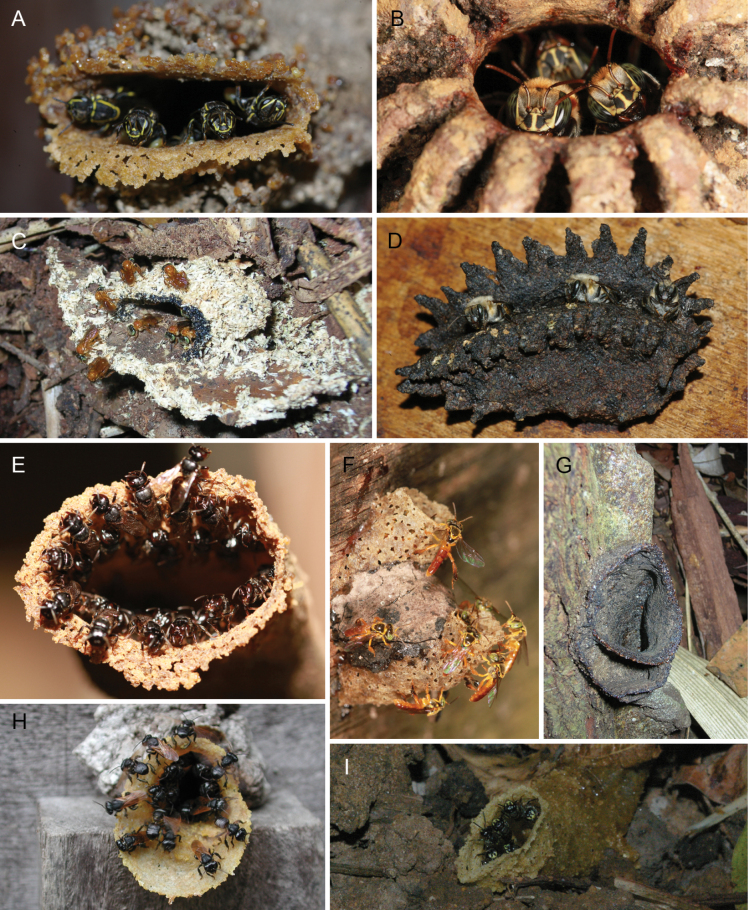
Nest entrances of representative stingless bees. **A***Paratrigona* sp. from Colombia **B**Melipona (Melikerria) grandis Guérin-Méneville from Peru **C**Tetragonula (Tetragonula) melanocephala (Gribodo) from Malaysia **D**Melipona (Michmelia) cf. eburnea Friese from Colombia **E***Lestrimelitta* sp. from Brazil **F***Tetragoniscaangustula* (Latreille) from Peru **G**Tetragonula (Tetragonula) sarawakensis (Schwarz) from Malaysia **H**Scaptotrigona (Astegotrigona) mexicana (Guérin-Méneville) from Mexico **I** stingless bee from Kenya. All photographs C. Rasmussen except H R. Ayala.

The nests of stingless bees are more intricate than any other bees and represent a cornucopia of interconnected layers and structures, most of which are composed of cerumen (Wille and Michener 1973) (Figs 5–7). Cerumen is a substance produced by the bees through the combination of wax, secreted from the bees’ tergal glands, with resin or other plant substances (plant resins stored by the bees are called propolis), or even enzymatic secretions (that is, some may add significant saliva) (Roubik 2023). Depending on the part of the nest, the cerumen may be further mixed with or bolstered by mud, feces, small pebbles, and/or sand. Mud and clay mixed with propolis by the bees is frequently referred to as geopropolis. Cerumens vary considerably, not only among bees, but for different parts of the nest and may therefore range from pliable to friable to exceedingly tough (Roubik 2023). Perhaps not surprisingly, tougher cerumen is often found toward the exterior, while more flexible materials, if present, are internal to the nest. The cerumen also embodies considerably antimicrobial properties, the result of both the bee secretions incorporated into the admixture or bestowed by the different plant resins (Shanahan and Spivak 2021; Roubik 2023). Thus, the chemical properties of the cerumen itself represents a form of colony defense from the most unseen of pathogenic invaders. An extensive line of research awaits comparative studies of the microbiomes of the unharvested resins, the unadulterated wax and bee secretions, the bees’ intestines, and the resulting cerumen used in different parts of the nest.

Nests vary widely but can be generally summarized as follows (Fig. 5): At the center is a brood chamber composed of brood cells. Individual brood cells are oriented horizontally (except vertically in *Dactylurina* and Scaura (Scaura) longula; Darchen and Pain 1966; Nogueira-Neto 1992), house a single developing bee, and open upward when the adult emerges (Fig. 6F). Mass provisions are provided before the queen lays an egg within the brood cell, which is thereafter sealed and left undisturbed until the new adult chews her way out. These cells are not reused and are broken down once development is completed. The brood cells are arranged in characteristic patterns, many times in a spiral pattern to form a horizontal comb (Fig. 6B, E), although in some species the cells are more irregularly placed in clusters, sometimes quite loosely (Fig. 6C, D). Brood clusters are most often found in those species of smaller sizes that can build nests within irregular cavities, interconnected cavities, or tight crevices that otherwise do not lend themselves to the more rigid spatial parameters required by stacks of horizontal combs. This brood chamber is wrapped by an involucrum (Fig. 5), often made of several tightly adjoined layers and thus, laminate (Roubik 2006). The involucrum maintains a stable temperature for the brood chamber and is important for thermoregulation in many species (Roubik 2006). Outside of the brood chamber, and outside of the involucrum when present, is the storage area (Fig. 5). Within this space the bees construct pots in clusters or in layers, but not distinct combs (Fig. 6C, G). These pots are built with cerumen and used to store pollen and honey. Surrounding the storage space, and thus enveloping the entirety of the nest, are layers of batumen (Fig. 5). The batumen layers, which are wall-like layers of particularly thick and cured resin. The outer layer of batumen can nicely fit against and within irregular surfaces or spaces. When the batumen is used to close off the nest from the remainder of a larger cavity, the layers are thick and tough, concrete-like (Fig. 5), and referred to as geopropolis. Similar tough layers of batumen are used to form the outer walls of exposed nests. A tube is built that leads from the storage space through the layers of batumen to the exterior of the nest. Entrance tubes can often be unique, even diagnostic for given genera, subgenera, or species, and range from simple, thin tubes with a singular opening to elaborate branched or multi-opening structures (Fig. 7).

**Figure 8.**
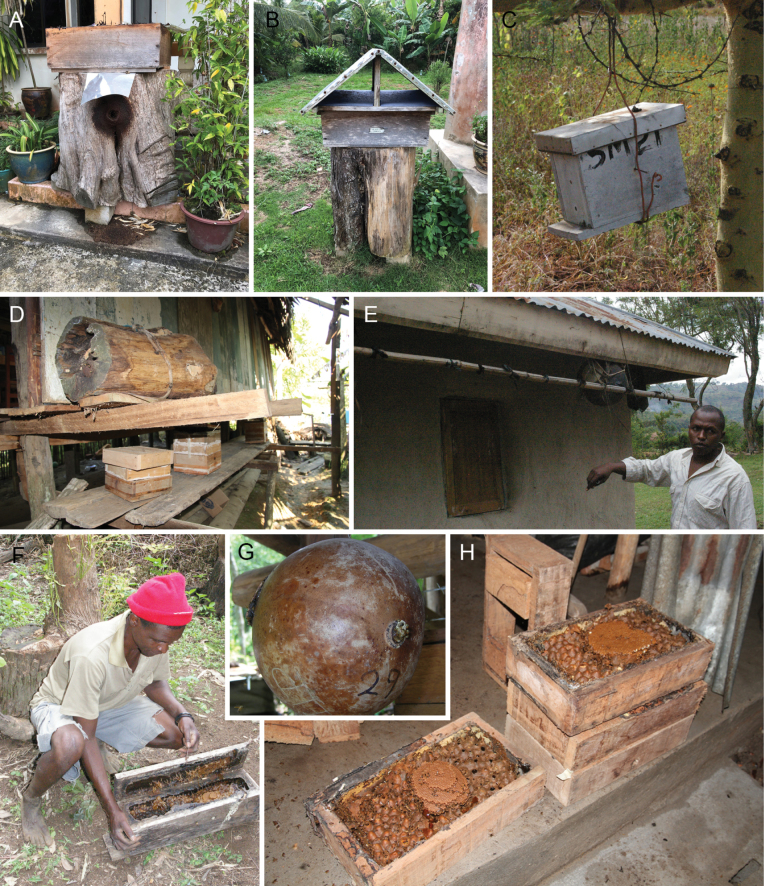
Managed stingless bee nests **A***Geniotrigonalacteifasciata* (Cameron) from Brunei **B***Lepidotrigonaterminata* (Smith) from Brunei **C** managed stingless bee colony from Kenya **D** stingless bee hives both in log and in boxes **E** stingless bee colonies kept in bamboo under the roof in Kenya **F** stingless beekeeper in Kenya **G** small colony of stingless bees kept in dried gourd in Peru **H***Scaptotrigona* sp. kept in boxed hive in Peru. All photographs C. Rasmussen except A and B M.S. Engel.

**Figure 9.**
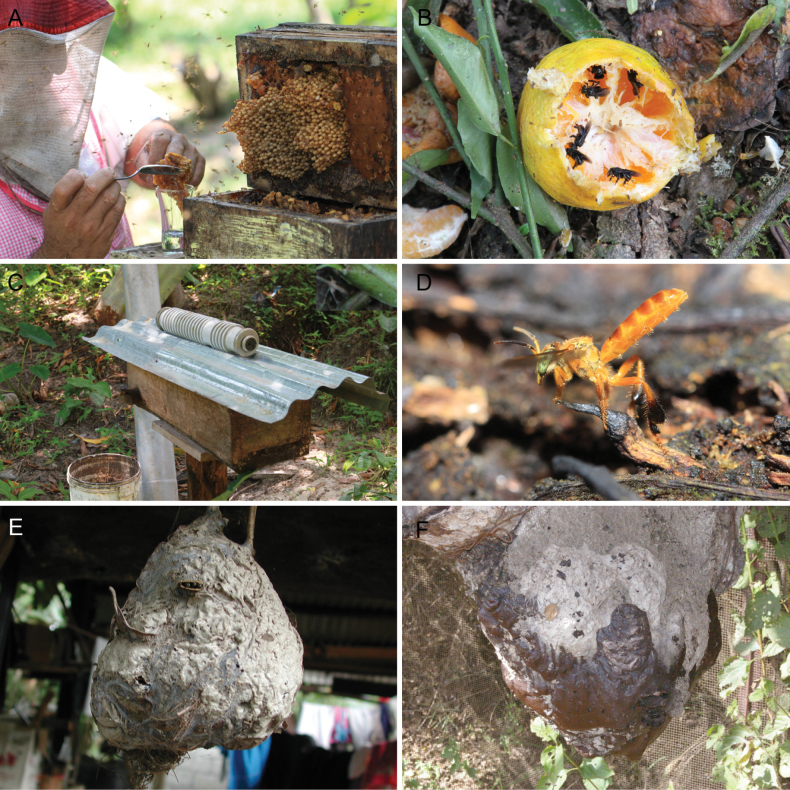
Managed and natural nests of stingless bees as well as workers collecting materials **A** beekeeper sampling comb and honey from a nest of *Frieseomelitta* sp. in Peru **B** workers of *Trigona* sp. collecting from a rotting orange in Peru **C** a managed nest of *Melipona* sp. in Peru **D***Tetragona* sp. [likely *Tetragonagoettei* (Friese)] grabbing a bit of pitch in Peru **E** arboreal nest of *Partamona* sp. in Mexico **F** arboreal nest of *Dactylurina* sp. in Kenya. All photographs C. Rasmussen except E R. Ayala.

Nest sites are a limiting factor for most bees and the same seems to be true for stingless bees. Indeed, this may have also been a contributing factor leading to the miniaturization bottleneck for Meliponini. Many species build nests within pre-existing cavities in tree trunks or branches, with a preference for spaces that have narrowed openings with the surrounding environment, and which can be easily closed by the bees during nest construction (Wille and Michener 1973; Roubik 2006). The tree hollows used are often the result of some decay, which perhaps has also fueled the use of antimicrobially active cerumen for the bee colonies. Other forms of cavities may also be used, such as hollows in the ground, spaces within cliff faces, and in a wide range of human-produced spaces: in the walls or roofs of homes, in pipes, in electrical boxes, or even within furniture. Other stingless bees build their nests inside the exposed aerial nests of ants or termites, building an entrance tube on the exterior of the host nest and then gradually excavating into the side of the host and constantly enclosing the growing space so as to prevent their “hosts” from entering the newly founded bee nest (Camargo 1984). At the other end of the spectrum, some species of *Partamona* will construct their own “cavities” by building out from a starting point, usually against a tree or cliff, while some species of *Trigona* do similarly by wrapping batumen walls around a tree branch (semi-exposed nests) (Camargo and Pedro 2003; Rasmussen and Camargo 2008). Only two stingless bees, of all Apoidea, are known to nest in a single kind of plant, or have a host melittophyte. They are western Amazonian *Plebeia* that live in the upper branches of large *Cecropia* (Roubik 2021). A diversity of nests, both natural and managed, are illustrated in Figs 8, 9.

Although stingless bees have an atrophied (vestigial) sting and lack an ability to sting it does not make them defenseless. They exhibit different mechanisms for protecting their nests, ranging from camouflaging the nest entrance, nest construction in places difficult to access, to an active defensive behavior, sometimes quite elaborate. They can tangle themselves in hair and fur, pinching the skin of the aggressor or intruder with their jaws, which can even cause some injuries, enter the nostrils, and ears of intruders, as well as depositing plant resins or caustic substances on the intruder, this last specialized behavior observed with the mandibular glands of bees in the genus *Oxytrigona* (it also has to be wondered if *Papuatrigona* has similarly caustic mandibular secretions: MSE, pers. obs.). If grabbed, many stingless bees will bend the metasoma around to their attacker, mimicking the behavior of stinging, perhaps relying on a Batesian-like mimicry with stinging bees and wasps. Lastly, some species, such as *Frieseomelittasilvestrii* (Friese), the bees will play dead (thanatosis) when they encounter a large enemy (Ihering 1912; Michener 1974).

## Supplementary Material

XML Treatment for Adactylurina

## Footnotes

* Scaptotrigona silviae Engel is potentially a junior synonym of S. marialiceae Laroca & Almeida, while S. hylaeana Nogueira & Santos-Silva is a synonym of S. tubiba (Smith) (MSE, pers. obs.).

## ﻿Additional information

### Author ORCIDs

Michael S. Engel https://orcid.org/0000-0003-3067-077X

Claus Rasmussen https://orcid.org/0000-0003-1529-6548

Ricardo Ayala https://orcid.org/0000-0002-7718-1853

Favízia F. de Oliveira https://orcid.org/0000-0003-4366-5005

### Data availability

All of the data that support the findings of this study are available in the main text or Supplementary Information.
